# Accumulation of TERT in mitochondria exerts two opposing effects on apoptosis

**DOI:** 10.1002/2211-5463.13682

**Published:** 2023-08-08

**Authors:** Hiroshi Ebata, Tomohiro Shima, Ryo Iizuka, Sotaro Uemura

**Affiliations:** ^1^ Department of Biological Sciences, Graduate School of Science The University of Tokyo Japan; ^2^ Present address: Buck Institute for Research on Aging Novato CA USA

**Keywords:** apoptosis, live‐cell imaging, mitochondria, oxidative stress, TERT

## Abstract

Telomerase reverse transcriptase (TERT) is a protein that catalyzes the reverse transcription of telomere elongation. TERT is also expected to play a non‐canonical role beyond telomere lengthening since it localizes not only in the nucleus but also in mitochondria, where telomeres do not exist. Several studies have reported that mitochondrial TERT regulates apoptosis induced by oxidative stress. However, there is still some controversy as to whether mitochondrial TERT promotes or inhibits apoptosis, mainly due to the lack of information on changes in TERT distribution in individual cells over time. Here, we simultaneously detected apoptosis and TERT localization after oxidative stress in individual HeLa cells by live‐cell tracking. Single‐cell tracking revealed that the stress‐induced accumulation of TERT in mitochondria caused apoptosis, but that accumulation increased over time until cell death. The results suggest a new model in which mitochondrial TERT has two opposing effects at different stages of apoptosis: it predetermines apoptosis at the first stage of cell‐fate determination, but also delays apoptosis at the second stage. As such, our data support a model that integrates the two opposing hypotheses on mitochondrial TERT's effect on apoptosis. Furthermore, detailed statistical analysis of TERT mutations, which have been predicted to inhibit TERT transport to mitochondria, revealed that these mutations suppress apoptosis independent of mitochondrial localization of TERT. Together, these results imply that the non‐canonical functions of TERT affect a wide range of mitochondria‐dependent and mitochondria‐independent apoptosis pathways.

## Graphical abstract

The mitochondrial accumulation of TERT has two opposite effects at different stages of stress‐induced apoptosis: it predetermines apoptosis at the first stage of cell fate determination but slows down the process at the later stage. Cells without TERT accumulation at the mitochondria can survive after stress stimuli, but when they die, they undergo rapid apoptosis.

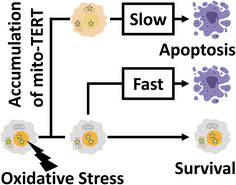

**Abbreviations**
FBS: fetal bovine serum
KDE: kernel density estimation
MCC: Manders' colocalization coefficient
MTS: mitochondrial targeting signal
RdRP: RNA‐dependent RNA polymerase
SPC: sodium percarbonate
TERT: telomerase reverse transcriptase

Telomerase reverse transcriptase (TERT) is a protein subunit of the telomerase complex, which elongates the telomeric repeat sequences at chromosomal ends and prevents telomere loss [1]. In humans, most cancer cells express high levels of TERT, whereas normal cells suppress TERT expression [2]. Since TERT increases the number of cell divisions, it is believed to promote the unlimited growth of cancer cells. Interestingly, several studies have shown that TERT is localized not only in the nucleus but also in mitochondria, which lack telomeric regions [3, 4, 5]. Oxidative stress, which eventually leads to cell apoptosis, has been reported to increase TERT localization in mitochondria [4, 5], raising the possibility of a non‐canonical role of mitochondrial TERT in apoptosis beyond its telomere elongation function. Mutagenesis studies have proposed that mitochondrial TERT induces apoptosis [4, 6]. However, TERT overexpression has been reported to increase TERT protein level in mitochondria and cell survival after oxidative stress, suggesting that mitochondrial TERT suppresses apoptosis [5, 7]. These reports, while conflicting, have shed light on the possibility that mitochondrial TERT regulates apoptosis.

These contradictory observations can be attributed mainly to three reasons. First, the relationship between TERT localization and cell death in individual cells has not been fully tested. Due to fluorescently labeled TERT failing to retain normal TERT enzymatic activity and cellular distribution, previous studies have only detected mitochondrial TERT after cell fixation or cell disruption [4, 5, 6]. Therefore, the eventual fate of each individual cell is unknown. Secondly, there is little temporal information. Previous immunofluorescence‐ and flow cytometry‐based measurements only provide information on cell death and TERT localization at specific times. However, oxidative stress induces cell death through several different pathways [8, 9]. Even within the same pathway, the response time to cell death‐inducing stimuli varies among cells. Thus, directly testing the relationship of mitochondrial TERT and cell death requires tracking the two factors over time. Thirdly, classical experimental methods can damage the cells. Immunofluorescence, for example, requires multiple washes for each step of fixation, permeabilization, and antibody binding. Cells undergoing cell death lose their adhesiveness to the dish surface, resulting in some being lost by the multiple washes. The loss of dead cells results in underestimating apoptosis. In flow cytometry measurements, flowing cells are exposed to physical stress including high pressures, shear forces, electrical charges, shock forces, and rapid temperature changes. These additional stresses can induce stress responses by the cells, resulting in more cell death. Therefore, directly testing the relationship between mitochondrial localization and cell death using stress‐free methods is needed.

In response, here we combined imaging‐based dead cell detection methods with live‐cell fluorescence imaging of TERT to directly assess the relationship between the mitochondrial localization of TERT and apoptosis of individual cells. Live‐cell imaging provides spatial and temporal information of the TERT distribution until cell death. This approach has several advantages over conventional dead cell detection methods. Namely, live‐cell imaging avoids extra physical stress to the cells because it requires only one medium exchange to cause oxidative stress without any washing steps. Also, to visualize TERT distribution in a cell, we fused TERT to a fluorescent protein, mVenus, at a position that did not interfere with the mitochondrial localization or enzymatic activity. This setup enabled us to track the temporal changes of TERT localization. Consequently, we found that the accumulation of TERT in mitochondria emerged immediately after oxidative stress in a subset of cells to cause apoptosis but, at the same time, the accumulation positively correlated with a longer time until cell death. These results suggest that mitochondrial TERT plays distinct roles at different stages of apoptosis. We also elucidated the effects of previously reported mutations in TERT and found that they are independent of TERT localization in mitochondria.

## Results

### Effects of TERT mutations R3E/R6E and Y707F on sub‐cellular distribution of TERT in HeLa cells

**Fig. 1:**
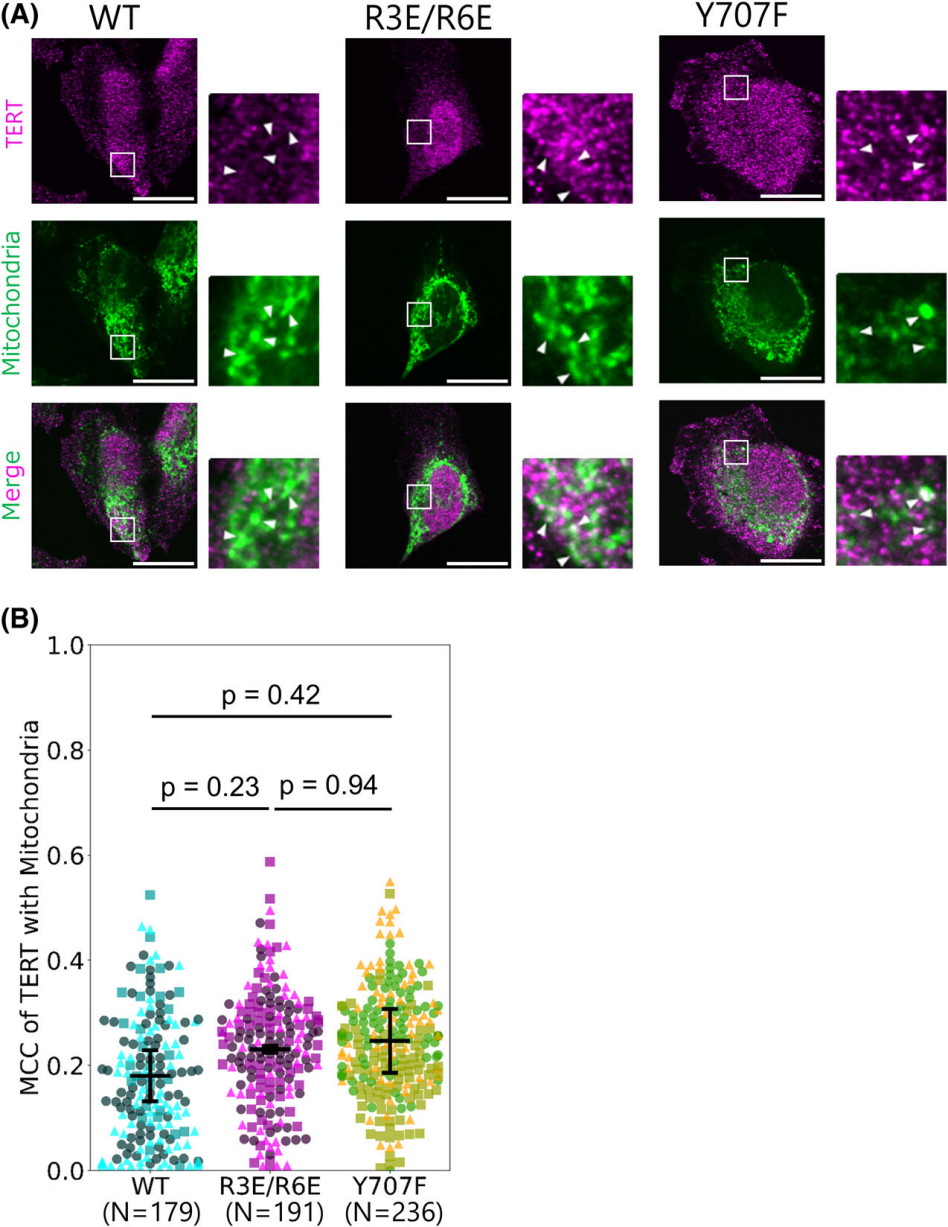
Effects of TERT mutations R3E/R6E and Y707F on sub‐cellular distribution of TERT in HeLa cells. (A) Representative immunofluorescence images of HeLa cells expressing the TERT constructs. Higher magnification images of the boxed areas are shown on the right. White arrowheads indicate fluorescent foci showing the overlap between TERT and mitochondria signals. Magenta, anti‐TERT immunofluorescence; green, MitoTracker Deep Red FM fluorescence. Scale bars, 20 μm. (B) Quantification of the mitochondrial localization of TERT. Manders' colocalization coefficient (MCC) of TERT with mitochondria was calculated from the fluorescence intensity of TERT and mitochondria. Dots show MCC of each cell and bars show the mean ± 95% confidence interval (C.I., 1.96 standard error of the mean (SEM)) from three independent experiments. Different markers represent different experiments. Wild‐type (WT), 179 cells; R3E/R6E, 191 cells; Y707F, 236 cells. Steel‐Dwass test was performed with a significance level of 0.05.

We first engineered TERT mutants and assessed their and wild‐type TERT localization to mitochondria. We prepared stable HeLa cell lines expressing wild‐type TERT (hereafter referred to as TERT_WT_) and TERT mutants using the Sleeping Beauty system [10]. The R3E/R6E mutation has been reported to inhibit the mitochondrial translocation of TERT due to the location of these residues in the mitochondrial targeting signal (MTS) of TERT [4]. Y707F has been reported to inhibit the oxidative stress‐stimulated nuclear export of TERT, which is dependent on phosphorylation of the tyrosine residue by Src kinases [6]. We examined the localization of these mutants by immunofluorescence of the cells cultured under normal conditions (no oxidative stress, Fig. 1A). Even though we also conducted immunofluorescence under oxidative stress, the majority of the cells were detached from the dish surface by the sample preparation steps, and thus, we did not include the immunofluorescence data under oxidative stress. The degree of TERT mitochondrial localization was quantified by Manders' colocalization coefficient (MCC) from the immunofluorescence images of these cell lines. MCC is an intuitive and direct metric measuring the co‐occurrence of the quantity of interest [11]. MCC of R3E/R6E and Y707F TERT mutants (hereafter referred to as TERT_R3E/R6E_ and TERT_Y707F_, respectively) and mitochondria approximated that of TERT_WT_ (Fig. 1B). We also quantified the TERT protein levels in mitochondria from these cell lines (Fig. S1) and found that neither R3E/R6E nor Y707F mutation showed the marked decrease in mitochondrial TERT protein levels, contrary to previous reports [4, 6]. A similar trend was observed in crude matrix samples in which mitochondria were further fractionated by the sodium carbonate treatment, suggesting that TERT is transported into interior of mitochondria in these cells (Fig. S1). The western blotting showed the TERT signals mainly at ~ 120 kDa, as expected from its molecular weight. However, we cannot conclude the amount of TERT protein in the bands, because mass spectrometry analysis of the bands did not detect any TERT‐derived peptides. This result is likely due to the absence of TERT in the bands beyond the detection limit of our mass spectrometry.

### TERT mutations R3E/R6E and Y707F increase cell survival

**Fig. 2:**
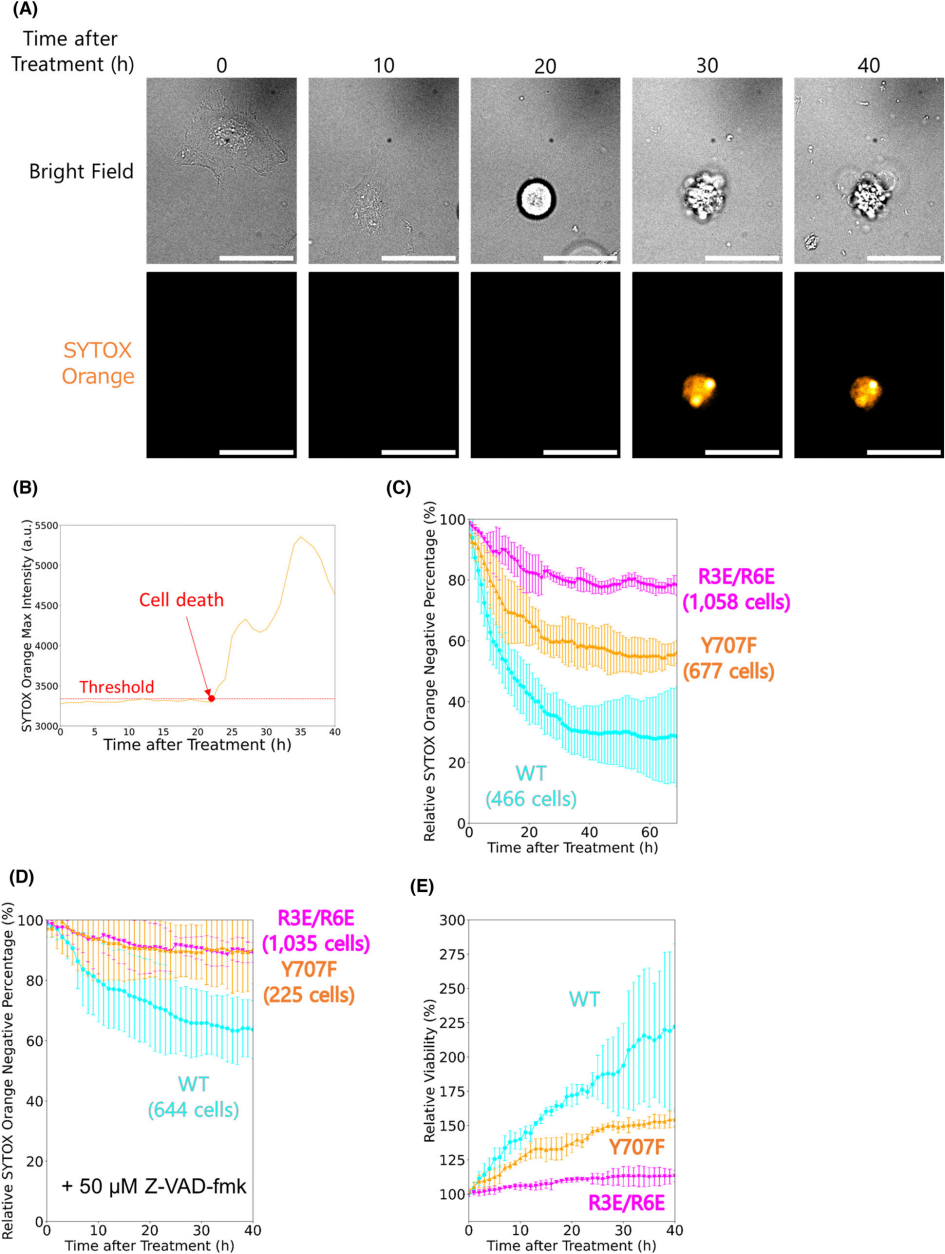
TERT mutations R3E/R6E and Y707F increase cell survival. (A) Representative live‐cell images of HeLa cells expressing the TERT constructs. Cells were treated with 267 μm SPC for 3 h before imaging. Orange, SYTOX Orange fluorescence. Scale bars, 50 μm. (B) A representative time‐course trace of the maximum fluorescence intensity of the dead cell staining dye SYTOX Orange from the cell depicted in (A). Cells were determined as positive after the SYTOX Orange fluorescence intensity exceeded a threshold (red dashed line). (C) Percentage of SYTOX Orange‐negative cells treated with 267 μm SPC for 3 h. All percentages were normalized to control experiments without oxidative stress (Fig. S2D). Error bars show 95% C.I. (1.96 SEM) from three independent experiments. WT, 466 cells; R3E/R6E, 1058 cells; Y707F, 677 cells. (D) Percentage of SYTOX Orange‐negative cells treated with 267 μm SPC for 3 h when 50 μm Z‐VAD‐fmk was added. All percentages were normalized to control experiments without oxidative stress (Fig. S2D). Error bars show 95% C.I. (1.96 SEM) from three independent experiments. WT, 644 cells; R3E/R6E, 1035 cells; Y707F, 225 cells. (E) Relative viability from percentages of SYTOX Orange‐negative cells in the presence of Z‐VAD‐fmk treated with 267 μm SPC for 3 h. The viability was calculated from the normalization of the percentages in the experiments with Z‐VAD‐fmk (D) to the percentages in the experiments without Z‐VAD‐fmk (C). Error bars show 95% C.I. (1.96 SEM) from 3 independent experiments.

Next, to observe the effect of the TERT mutations on the apoptotic response, we introduced oxidative stress to the cells and tracked each cell by live‐cell imaging under a fluorescence microscope (Fig. 2A). Conventionally, oxidative stress treatment requires the omission of fetal bovine serum (FBS) in the medium since FBS scavenges H_2_O_2_ and attenuates the effect of oxidative stress. However, even when we cultured cells in the medium without oxidative stress and FBS, the majority of cells were detached from the dish surface after medium exchange. In addition, even with FBS, oxidative stress treatment caused approximately 70% of the cells expressing TERT_WT_ to undergo cell death. Thus, we decided to add FBS in culture medium even during oxidative stress treatment in this work (see ‘Dead cell detection with live‐cell imaging’ in Materials and methods). We determined the time until cell death by the fluorescence intensity of SYTOX Orange, which stains dead cells, and YO‐PRO‐1, which stains apoptotic cells (Fig. 2B and Fig. S2A, see ‘Microscopy for live‐cell imaging and tracking’ in Materials and methods). Survival curves of these cell lines demonstrated that HeLa cells expressing either TERT mutant were more resistant to oxidative stress than those expressing TERT_WT_ (Fig. 2C). This result agrees with previous studies that reported increased cell viability in the mutants after oxidative stress [4, 6]. In all conditions, the cell survival percentage reached a plateau at approximately 40 h after oxidative stress treatment. Finally, the survival curve of the SYTOX Orange signal lagged that of the YO‐PRO‐1 signal by several hours (Fig. S2B). Of note, all the survival percentages were normalized to the data from control cells in the medium without oxidative stress (Fig. S2C–E).

To determine whether apoptosis inhibition changes the survival curves, we did the same experiments using a pan‐caspase inhibitor Z‐VAD‐fmk [12]. Z‐VAD‐fmk increased the survival of all HeLa cell lines (Fig. 2D), but the extent of the increase differed among the lines (Fig. 2E). Drastic changes in the viability of cells expressing TERT_WT_ by Z‐VAD‐fmk indicated that cell death induced by oxidative stress was predominantly apoptosis, as previously reported [13]. Z‐VAD‐fmk increased the viability at 40 h after oxidative stress treatment by approximately 120% in HeLa cells expressing TERT_WT_, 50% in cells expressing TERT_Y707F_, and only 10% in cells expressing TERT_R3E/R6E_ (Fig. 2E). These results indicate that the caspase inhibition effect of Z‐VAD‐fmk was blunted in cell lines expressing the TERT mutants, supporting the notion that these mutations inhibit the apoptotic process even without Z‐VAD‐fmk. Collectively, the lag of the SYTOX Orange signal compared with the YO‐PRO‐1 signal and the increase in cell survival by Z‐VAD‐fmk suggest that apoptosis is the predominant cell‐death pathway of HeLa cells overexpressing TERT_WT_ under oxidative stress and that the mutations inhibit apoptosis.

### Probing TERT without interfering with its activity or mitochondrial localization

**Fig. 3:**
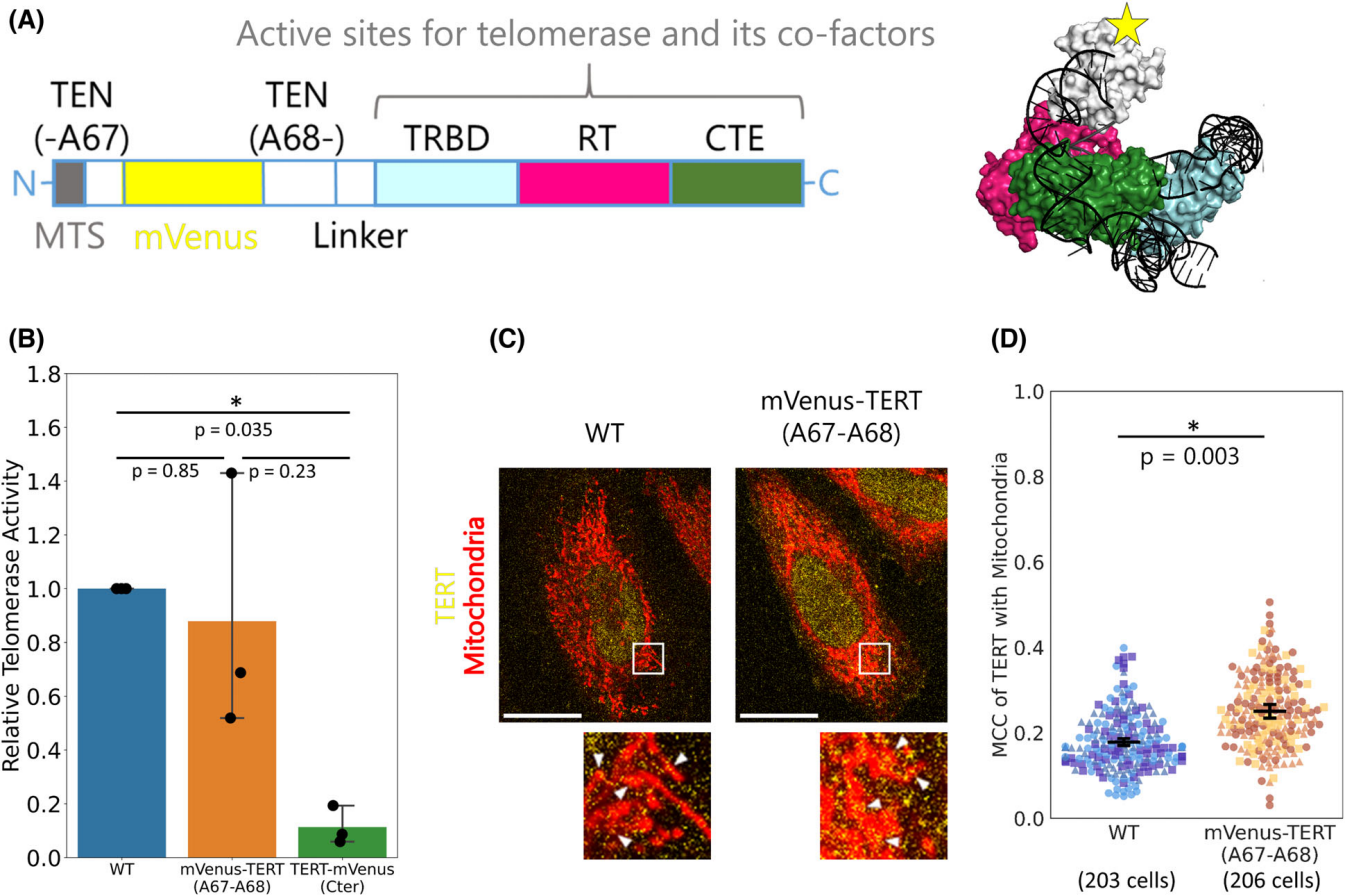
TERT was probed without interfering with its activity or mitochondrial localization. (A) Schematic representation of mVenus‐TERT for live‐cell tracking. The structural model was created from the cryo‐EM structure data (Electron Microscopy Data Bank accession number EMD‐7518). MTS, mitochondrial targeting signal; TEN, telomerase essential N‐terminal domain; TRBD, telomerase RNA‐binding domain; RT, reverse transcriptase domain; CTE, C‐terminal extension. (B) Telomerase activity of whole‐cell extracts from HeLa cells expressing the TERT constructs measured by a qPCR‐based telomerase activity assay. TERT‐mVenus (Cter) is a TERT construct conjugated with mVenus at its C terminus. Dots show individual data points and bars show the mean ± 95% C.I. (1.96 SEM) from three independent experiments. Steel‐Dwass test was performed with a significance level of 0.05 (*). (C) Representative immunofluorescence images of HeLa cells expressing the TERT constructs. Higher magnification images of the boxed areas are shown on the right. White arrowheads indicate fluorescent foci showing the overlap between TERT and mitochondria signals. Yellow, anti‐TERT immunofluorescence; red, MitoTracker Deep Red FM fluorescence. Scale bars, 20 μm. (D) Quantification of the TERT localization. MCC of TERT with mitochondria was calculated from the TERT and mitochondria fluorescence intensities. Dots show MCC of each cell and bars show the mean ± 95% C.I. (1.96 SEM) from three independent experiments. Different markers represent different experiments. WT, 203 cells; mVenus‐TERT (A67‐A68), 206 cells. Mann–Whitney *U*‐test was performed with a significance level of 0.05 (*).

Next, to directly investigate the relationship between the mitochondrial localization of TERT and apoptosis in individual cells, we inserted a fluorescent tag mVenus into TERT. In preparation for the visualization of TERT localization by live‐cell tracking, we assessed the effect of the insertion on the telomerase activity and localization pattern of TERT. Conventionally, epitope tags or fluorescent proteins are conjugated to TERT at the N or C terminus [6, 14, 15]. However, TERT has an MTS at the N terminus and an essential domain for its telomerase enzymatic activity at the C terminus [3, 16, 17], suggesting conjugation at either location could affect TERT function. Moreover, a previous report suggested that mitochondrial TERT functions dependent on its reverse transcriptase activity associated with mitochondrial DNA and tRNAs [18], which is consistent with other reports that TERT can bind to mitochondrial nucleotides [19, 20]. Therefore, we inserted mVenus between A67 and A68 (hereafter referred to as mVenus‐TERT), which we considered an ideal location because it is far from the active sites for the telomerase complex and telomerase co‐factors (Fig. 3A) [21, 22]. We evaluated the telomerase activity and localization of mVenus‐TERT_WT_ by qPCR‐based telomerase activity assay and immunofluorescence (Fig. 3B–D). As expected, mVenus insertion did not interfere with the telomerase activity, while conjugating mVenus to the C terminus (TERT‐mVenus) deteriorated telomerase activity (Fig. 3B). Of note, the difference in the telomerase activity among these cell lines was not a consequence of different TERT protein expression levels (Fig. S3). mVenus‐TERT_WT_ showed a higher mitochondrial localization, which might result from inhibiting nuclear import of TERT, but at least, mVenus insertion did not decrease mitochondrial localization of TERT. Accordingly, we used mVenus‐TERT to visualize TERT during apoptosis by live‐cell imaging.

### Simultaneous live‐cell tracking of cell death and TERT localization

**Fig. 4:**
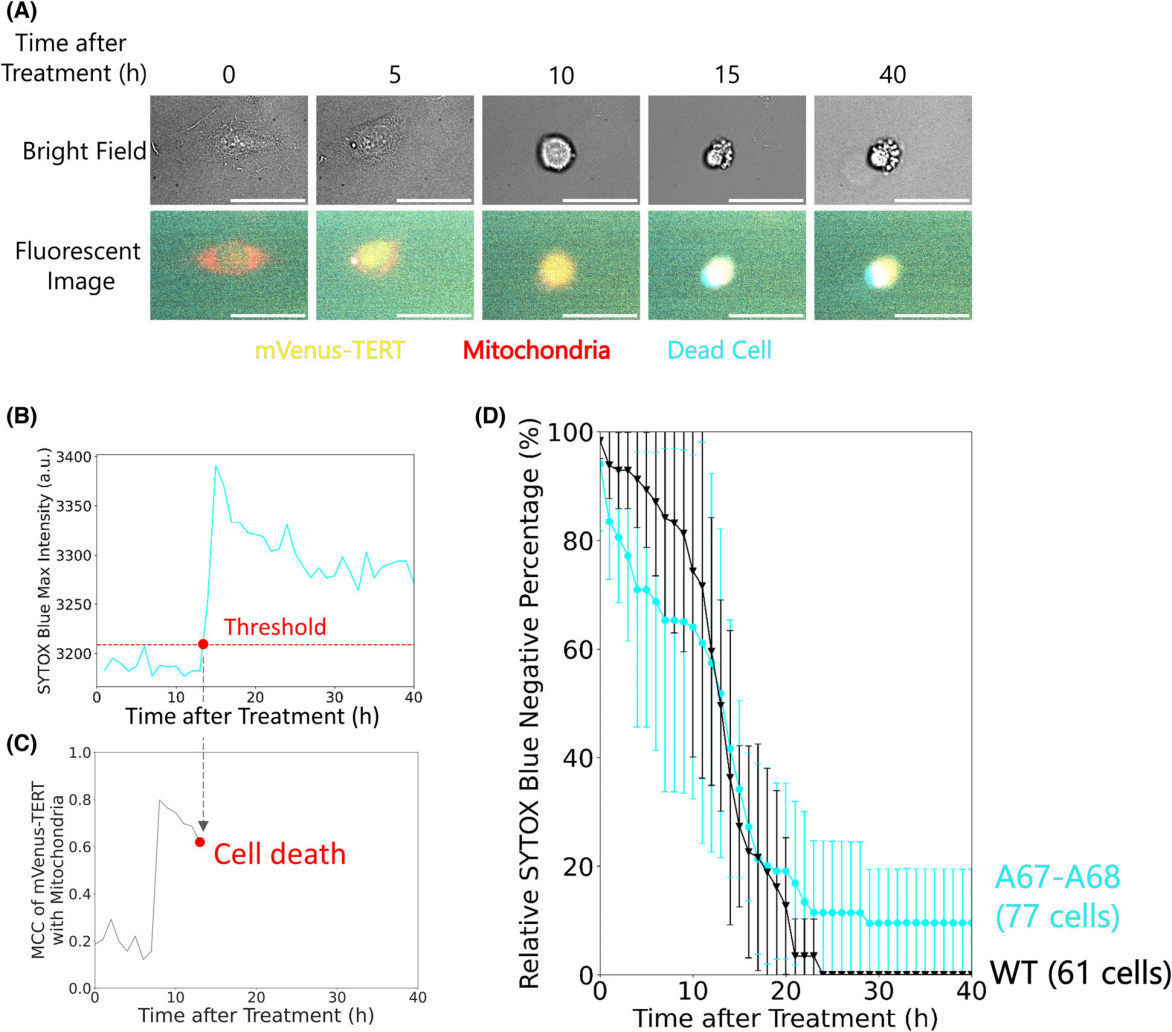
Simultaneous live‐cell tracking of cell death and TERT localization. (A) Representative live‐cell images of HeLa cells expressing mVenus‐TERT constructs. Yellow, mVenus fluorescence; red, MitoTracker Deep Red FM fluorescence; cyan, SYTOX Blue fluorescence. Cells were treated with 267 μm SPC for 3 h before the imaging. Scale bars, 50 μm. (B) Representative time‐course traces of maximum SYTOX Blue fluorescence intensity of the cell shown in (A). Cells were determined as positive after the SYTOX Blue fluorescence intensity exceeded the threshold. (C) Representative time‐course traces of MCC of mVenus‐TERT with mitochondria of the cell in (A). (D) Percentages of SYTOX Blue‐negative cells treated with 267 μm SPC among cells expressing wild‐type TERT or mVenus‐TERT (A67‐A68). All percentages were normalized to control experiments without oxidative stress (Fig. S4B). Error bars show 95% C.I. (1.96 SEM) from three independent experiments. WT, 61 cells; mVenus‐TERT (A67‐A68), 77 cells.

In addition to evaluating telomerase activity and localization, we compared the survival of cells harboring TERT_WT_ and mVenus‐TERT_WT_ to assess the possibility that mVenus compromises cell survival. We performed the following three simultaneous fluorescence measurements: dead cells visualized by SYTOX Blue staining, mitochondria visualized by MitoTracker Deep Red FM, and TERT visualized by mVenus (Fig. 4A). The time until apoptosis was calculated as the time when the fluorescence intensity of SYTOX Blue reached a specified threshold (see ‘Microscopy for live‐cell imaging and tracking’ in Materials and methods). Also, we calculated MCC of mVenus‐TERT with mitochondria from the fluorescence intensity at each time point (Fig. 4B,C). From the time until apoptosis, we found that cells expressing TERT_WT_ or mVenus‐TERT_WT_ had similar survival percentages after oxidative stress (Fig. 4D). This result showed that by optimizing the insertion location, it is possible to produce a fluorescently labeled TERT that retains not only its normal localization and activity but also normal cell‐death properties upon oxidative stress. Moreover, live‐cell imaging without oxidative stress did not show any cytotoxicity (Fig. S4). Therefore, we concluded that cell death after oxidative stress was caused mainly by stress and not by mVenus insertion.

### Live‐cell tracking revealed opposing effects of mitochondrial TERT in apoptosis

**Fig. 5:**
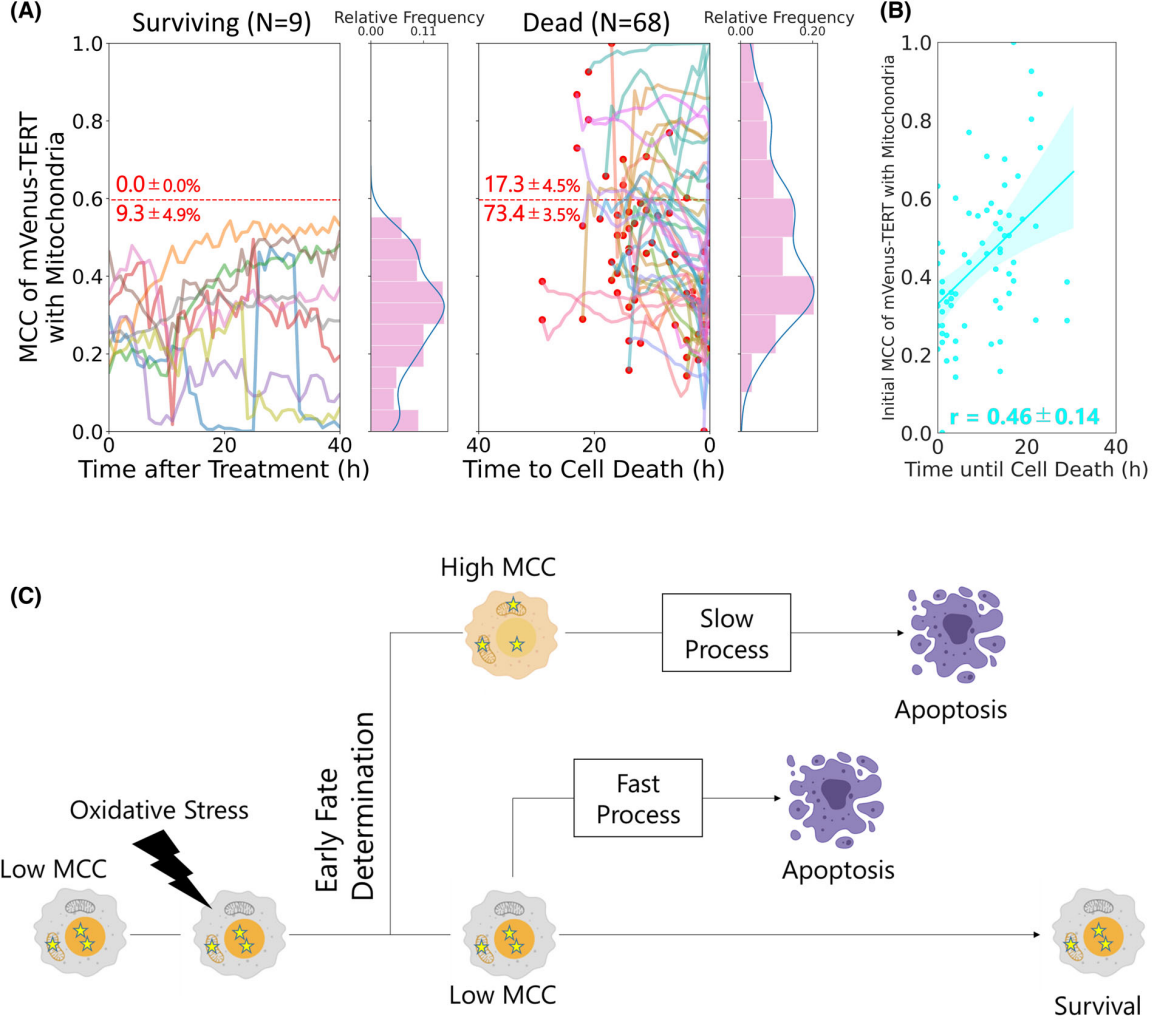
Live‐cell tracking reveals the effects of mitochondrial TERT in apoptosis. (A) Time‐course plot of MCC of mVenus‐TERT with mitochondria in each cell. A histogram and KDE plot of all MCCs in each group of surviving and dead cells are shown. As oxidative stress, we treated cells with 267 μm SPC for 3 h. Plotted values are the mean values per 5 frames. For dead cells, 0 in the *x*‐axis represents the moment the cells died. Red dots show MCC at the beginning of the observation. Red numbers show the percentage (mean ± SEM from three independent experiments) of cells whose MCC was above or below the threshold represented by the red dashed line. Surviving cells (Surviving), nine cells; Dead cells (Dead), 68 cells. (B) A scatter plot and regression line between the initial MCC of mVenus‐TERT in each dead cell and duration until cell death. Translucent bands around the regression line represent 95% C.I. (1.96 SEM). *r*, Pearson's correlation coefficient (PCC) shows the mean ± SEM from three independent experiments. Total 68 cells. (C) A new model for the roles of mitochondrial TERT in apoptosis. Mitochondrial TERT has two opposing effects at different stages of apoptosis. Mitochondrial accumulation of TERT (high MCC) predetermines apoptosis at the first stage of cell‐fate determination, whereas retention of TERT outside of the mitochondria (low MCC) does not yet make the decision to undergo apoptosis. In the second stage, cells with high MCC undergo delayed apoptosis, whereas apoptotic cells with low MCC execute rapid apoptosis. This figure was created with BioRender.com.

From the live‐cell tracking, we obtained the time‐course plot of the MCC of mVenus‐TERT with mitochondria after oxidative stress in cells expressing mVenus‐TERT_WT_. The histogram and kernel density estimation (KDE) plot of all MCCs from the time‐course plot demonstrated that cells with high MCC did not survive and all surviving cells showed low MCC (Fig. 5A). In contrast, cells cultured without oxidative stress did not show the cell population with high MCC in the KDE plot (Fig. S5). Apoptotic cells show unique morphological features such as shrunken cellular bodies, meaning high MCC could result from the false detection of nuclear TERT as mitochondrial TERT. Live‐cell tracking for the nuclear stain Hoechst 33342 demonstrated that cell death increased the MCC of Hoechst 33342 with mitochondria in the KDE plot, similar to the MCC of mVenus‐TERT with mitochondria (Fig. S6). However, unlike MCC of mVenus‐TERT, MCC of Hoechst 33342 did not show an additional peak at high values, verifying that the high MCC of mVenus‐TERT with mitochondria is not an experimental artifact and that TERT accumulates in mitochondria upon oxidative stress in a subset of cells. mVenus intensity showed the tendency of decreased TERT expression in dying cells under oxidative stress without reaching statistical significance (Fig. S7), which supports the qPCR data showing decreased TERT expression induced by oxidative stress from a previous study [23]. Additionally, we found a positive correlation between MCC at the start of the imaging and time until apoptosis in cells expressing mVenus‐TERT_WT_ (Fig. 5B). This observation implies that cells expressing TERT_WT_ with high MCC tend to take longer to undergo apoptosis. From these results, we established a model for the role of mitochondrial TERT in apoptosis (Fig. 5C).

### TERT mutants R3E/R6E and Y707F inhibit the death of cells with low MCC and delay apoptosis independent of TERT localization

We also evaluated the effects of the TERT mutations using our live‐cell tracking system. Similar to the live‐cell imaging of TERT_WT_, the TERT_R3E/R6E_ and TERT_Y707F_ mutants displayed the increased cell survival even if bound to mVenus (Fig. S8A). Also, the time course of MCC of mVenus‐TERT with mitochondria after oxidative stress in these cells showed that viability of cells with high MCC was lower than that of cells with low MCC (Fig. S8B). Except for one mVenus‐TERT_Y707F_ cell survived, all cells with high MCC died after oxidative stress. These high death percentages of cells with high MCC in mVenus‐TERT mutants is similar to what we observed in mVenus‐TERT_WT_ (Fig. 5A). However, unlike for mVenus‐TERT_WT_ (Fig. 5B), there was no correlation between the initial MCC and time until apoptosis in the cells expressing mVenus‐TERT mutants (Fig. S8C). Next, to visualize the difference between the cells with high or low initial MCC, we set a threshold at approximately 0.60 (red dashed lines in Fig. 5A and Figs S5, S6, and S8B), as only a small subset of surviving cells showed higher initial MCC than the threshold (0% for mVenus‐TERT_WT_, 0% for mVenus‐TERT_R3E/R6E_ and 1.5% for mVenus‐TERT_Y707F_). The percentages of survived cells among cells with low initial MCC appeared to vary among the cell lines (WT, 9.3%; R3E/R6E, 24.1%; Y707F, 25.6%); however, further experiments are needed to evaluate this trend in terms of frequency‐based statistics. Although the differences in percentages did not reach statistical significance (Fig. S8B; *P* = 0.06 for WT–R3E/R6E and *P* = 0.05 for WT–Y707F, Steel‐Dwass test with a significance level of 0.05), the statistical power (1 − *β*) of the test was only 0.33, indicating a high probability (0.67) of a false negative result of the test. Therefore, the possibility remains that the hypothesis that the differences are significant is falsely rejected due to the low statistical power of the test. *Post‐hoc* analysis suggested that more than twice the number of cells (*N* > 139 for each cell line) are required to increase the statistical power above 0.8 for determining if the differences among the cell lines were statistically significant at the standard confidence level. The time until apoptosis of cells with low MCC of mVenus‐TERT mutants was the same as those with high MCC of mVenus‐TERT mutants (Fig. S8D), explaining the lack of correlation between the initial MCC and time until apoptosis in cells expressing mVenus‐TERT mutants (Fig. S8C). In addition, the time until the apoptosis of cells expressing mVenus‐TERT mutants was equivalent to that of cells with high MCC of mVenus‐TERT_WT_ and longer than that of cells with low MCC of mVenus‐TERT_WT_. The time difference between mVenus‐TERT_WT_ and mVenus‐TERT mutants in cells with low MCC implies that the mutants delayed cell death induced by oxidative stress. Fitting the time‐course plots of MCC showed that TERT barely changed its localization after oxidative stress (Fig. S8E). Taken together, while the time difference between mVenus‐TERT_WT_ and mVenus‐TERT mutants did not reach statistical significance, the mutants showed inhibited and delayed effects on stress‐induced cell death in cells showing low MCC.

## Discussion

To reveal the role of mitochondrial TERT in apoptosis, it is essential to observe the relationship between the TERT distribution in each cell and cell fate. Here, we achieved this by combining live‐cell imaging‐based dead cell detection with the observation of a new mVenus‐TERT reporter, which retains TERT properties. Our simultaneous tracking of the cell‐death process and TERT distribution of individual cells demonstrated a strong correlation between a high mitochondrial accumulation of TERT and cell death, but the high accumulation was also positively correlated with a longer time until cell death. These results suggest that TERT localization in mitochondria plays distinct roles at different stages of apoptosis.

Based on these results, we propose a new model that integrates the seemingly contradictory data from previous studies for the role of TERT in apoptosis. We speculate that there are two stages after oxidative stress. In the first stage, which is immediately after oxidative stress, mitochondrial TERT promotes apoptosis. Live‐cell tracking of TERT_WT_ localization revealed that after oxidative stress, all cells with high MCC of mVenus‐TERT with mitochondria died, while all surviving cells showed lower MCC. This observation of the mitochondrial localization of TERT after oxidative stress is consistent with previous reports [4, 5]. In the present study, the cells experienced 3 h of oxidative stress before tracking, and cells with high MCC appeared in the first frame of the tracking. Therefore, cells that showed an accumulation of mitochondrial TERT had their fate determined within 3 h of the oxidative stress treatment. Because all the cells with high MCC died after oxidative stress in this study and other reports found mitochondrial TERT to induce apoptosis [4, 6], we propose a model in which this accumulation predetermines apoptosis.

However, in the second stage, mitochondrial TERT delays the apoptotic process. In cells expressing TERT_WT_, the initial MCC of mVenus‐TERT with mitochondria positively correlated with the time until apoptosis. This correlation indicates that more TERT in mitochondria delays apoptosis. This delay can explain why previous studies found that mitochondrial TERT suppresses apoptosis [5, 7]. Several reports suggest that TERT inhibits the mitochondrial pathway of apoptosis through the interaction with anti‐apoptotic protein Bcl‐2 such as by inhibiting the conformational activation of pro‐apoptotic Bcl‐2 family protein BAX [24, 25]. TERT has also been reported to have a conserved motif among Bcl‐2 family proteins, through which it interacts with the anti‐apoptotic Bcl‐2 family proteins Bcl‐xL and Mcl‐1 [26]. The live‐cell tracking method that we developed in this study has the potential to reveal the real‐time interaction between TERT and Bcl‐2 family proteins during apoptosis.

Both TERT mutations R3E/R6E and Y707F are thought to suppress apoptosis by inhibiting the mitochondrial translocation of TERT. We evaluated this possibility by live‐cell tracking. Similar to TERT_WT_, almost all cells with high MCC of mVenus‐TERT with mitochondria did not survive, demonstrating that these mutations did not completely inhibit the mitochondrial translocation of TERT. Without oxidative stress, the mutations did not inhibit the mitochondrial localization of TERT in immunofluorescence and western blotting, which are in contrast to previous reports [4, 6, 27]. However, mass spectrometry of the ~ 120‐kDa bands of the mitochondrial samples did not show any peptides derived from TERT. This could be caused by the relatively low sensitivity of our mass spectroscopy due to the smear bands in Coomassie Brilliant Blue staining, but also raises the possibility that the discrepancy in western blotting was due to the inefficient targeting of the anti‐TERT antibody. Moreover, we quantified the TERT localization in each cell differently from previous studies using a colocalization index, MCC, which could also lead to produce different outcomes in this study. Furthermore, we evaluated the localization of TERT_R3E/R6E_ in HeLa cells, which highly express TERT endogenously, whereas previous studies evaluated the localization of this TERT mutant in normal human fibroblasts and human diploid lung fibroblast MRC‐5 cell lines, both of which do not express TERT endogenously. Since TERT oligomerization has been reported [28], it is possible that in our study, endogenous wild‐type TERT oligomerized with exogenous TERT carrying R3E/R6E for transport into mitochondria. To test this hypothesis, knockout experiments of the TERT locus from HeLa cells would be helpful. In any case, our data suggest that TERT mutations R3E/R6E and Y7070F suppress apoptosis in cells without TERT accumulating in mitochondria. Therefore, we assume that these mutations suppress apoptosis independently of mitochondrial TERT accumulation.

Compared with mVenus‐TERT_WT_, mVenus‐TERT_R3E/R6E_ and mVenus‐TERT_Y707F_ suppressed and delayed apoptosis in the cells with low MCC, suggesting that these mutations alter the cell death capacity of nuclear or cytoplasmic TERT under oxidative stress. Regardless of its mitochondrial accumulation, TERT has been reported to act as a suppressor of apoptosis [29, 30]. The negative effects of the TERT mutations on stress‐induced cell death are likely due to such a role of TERT in pathways other than mitochondrial. For instance, besides its canonical function as a telomerase, TERT has been reported to serve as an RNA‐dependent RNA polymerase (RdRP) and to regulate siRNAs and miRNAs [31, 32]. In addition, TERT has also been reported to act as a transcription regulator through Myc [33, 34], Wnt/β‐catenin [33, 35], and NF‐κB [36] pathways. Especially, NF‐κB pathway regulates apoptosis‐related proteins such as Bcl‐2. R3E/R6E and Y707F might change the TERT capacity as a transcription regulator and the expression pattern of apoptosis‐related proteins, resulting in the blunted effect of Z‐VAD‐fmk to these mutants. The R3E/R6E mutation has been reported to result in the increased activity of autophagy [27]. The transcriptional change in autophagy pathway induced by R3E/R6E and Y707F could play a protective role under oxidative stress observed in our study. Therefore, transcriptome analyses of cells expressing the TERT mutants with and without oxidative stress will help understanding how the mutations suppress the apoptotic pathway.

Our new model of apoptosis regulation by mitochondrial TERT is mainly based on the negative correlation between the initial MCC with the survival rate and the positive correlation between the initial MCC and duration until cell death. We assume that the initial MCC is the causality of these correlations, but it is necessary to regulate mitochondrial localization of TERT completely to validate this assumption. In this study, we had expected mitochondrial localization suppression in R3E/R6E and Y707F, but these mutations did not display altered TERT localization. Therefore, further genetic engineering of TERT, especially in the localization signal sequence, will be needed to directly assess our model. Also, the comprehensive molecular pathway of apoptosis regulation by mitochondrial TERT remains elusive. One such mechanism would be mitochondrial injury including mitochondrial DNA damage and the decrease of mitochondrial membrane potential. TERT has been reported to relate to mitochondrial injury [4, 7, 19, 20], which plays an important role in apoptosis. To reveal how TERT impinges on this pathway, monitoring mitochondrial status and TERT localization as well as interactome and transcriptome analyses of cells after inducing mitochondrial injury are crucial. This study demonstrated that cells under oxidative stress enter different states at different timing. Our method can provide such cellular information in real‐time and can be applied to select cells in a particular state before conducting further analyses, and therefore, has a potential to become a base method for future studies on the molecular basis of mitochondrial TERT in apoptosis regulation.

The live‐cell tracking method that we developed in this study can be used for other purposes. For instance, applying this method to other cell lines besides HeLa cells will elucidate whether our finding is universal. In the current system, accurate subcellular imaging is limited to adherent cells, but combining live‐cell tracking with tiny microwells [37] or flow cytometry [38] will broaden the method to floating cells. Also, live‐cell tracking can be used to investigate the roles of mitochondrial TERT in DNA protection [4, 20], ROS production [4, 7, 19], autophagy [27], mitophagy [39], and cellular senescence [6, 40, 41]. Same as apoptosis studies, combining our method with interactome and transcriptome analyses will accelerate our understanding of how TERT participates in these phenomena and cell metabolism beyond its function as a telomerase.

## Materials and methods

### Plasmids

The gene encoding TERT was amplified from the plasmid pCDH‐3xFLAG‐TERT, which was a gift from Steven Artandi at Stanford Cancer Institute Core, Stanford University (Addgene plasmid #51631; http://n2t.net/addgene:51631). The mVenus sequence was isolated from pCS2‐mVenus plasmid, which was purchased from the RIKEN BioResorce Research Center (Ibaraki, Japan, RDB15116). pSBbi‐Pur was a gift from Eric Kowarz at Institute of Pharmaceutical Biology, Goethe‐University (Addgene plasmid #60523; http://n2t.net/addgene:60523). The gene encoding TERT and mVenus was inserted into pSBbi vector using the In‐Fusion cloning kit (Takara Bio, Shiga, Japan). The R3E/R6E mutation was introduced using the KOD mutagenesis kit (Toyobo, Osaka, Japan). The Y707F mutation was introduced by PCR. All plasmids were transformed into DH5α chemical competent cells and purified using the FastGene miniprep kit and endotoxin‐free miniprep kit (Nippon Genetics, Tokyo, Japan). The primer sets used are presented in Table S1.

### Cell culture and generation of stable cell lines

The HeLa cell line was purchased from the RIKEN BioResource Research Center (RCB0007). Cells with 80–100% confluency in 6‐well plates were transfected with 1.9 μg of pSBbi‐TERT‐Pur plasmid, 0.1 μg of pCMV‐SB100 [10] and 3.75 μg of polyethylenimine in 250 μL of Opti‐MEM (Thermo Fisher Scientific, Waltham, MA, USA). After transfection, cells were selected in DMEM (Thermo Fisher Scientific, 10569044) supplemented with 10% FBS, 1 × Penicillin–Streptomycin (PenStrep), and 1 μg·mL^−1^ Puromycin for 1 week. All cells were cultured in DMEM supplemented with 10% FBS and 1 × PenStrep in a 37 °C incubator at 5% CO_2_.

### Immunofluorescence

Two days before immunofluorescence, the cells were passaged in a black‐walled poly‐l‐lysine‐coated 96‐well glass bottom dish (Matsunami, Osaka, Japan, GP96001) at a density of 4000 cells per well. The cells were incubated with DMEM supplemented with 10% FBS, 1 × PenStrep, and 100 nm MitoTracker Deep Red FM (Thermo Fisher Scientific) for 30 min at 37 °C and 5% CO_2_. All procedures after incubation with MitoTracker Deep Red FM were performed at room temperature in the dark. Hereafter, the washing step refers to the exchange of the medium for phosphate‐buffered saline (PBS; Nacalai, Kyoto, Japan) and to incubating the cells for 5 min.

Fixation was performed using 4% paraformaldehyde in PBS for 10 min followed by the washing step. Permeabilization was performed with 0.5% Triton‐X‐100 in PBS for 10 min followed by three washing steps. Blocking was performed with 3% bovine serum albumin in PBS with 0.1% Tween 20 (PBS‐T) for 1 h followed by the washing step.

For immunofluorescence with the TERT mutations, the cells were incubated with 20 μm Cellstain Hoechst 33342 (Fujifilm Wako Pure Chemical Corporation, Osaka, Japan) in PBS for 10 min followed by the washing step. The cells were then incubated with anti‐TERT antibody (Rockland, Limerick, PA, USA, 600‐401‐252S, 1 : 500 dilution) in PBS‐T for 1 h followed by three washing steps. Finally, the cells were incubated with anti‐rabbit IgG (H + L), F(ab')_2_ fragment conjugated with Alexa Fluor 488 (Cell Signaling Technology, Danvers, MA, USA, 4412S) in PBS‐T for 1 h followed by three washing steps.

For the immunofluorescence of cells expressing mVenus‐TERT_WT_, the cells were incubated with anti‐TERT antibody conjugated with CF405M dye (1 : 100 dilution) in PBS‐T for 1 h followed by three washing steps. Dye conjugation was performed using the Mix‐n‐Stain CF405M Antibody Labeling Kit (Biotium, Fremont, CA, USA). Anti‐TERT antibody conjugated with CF405M was diluted at 1 : 5 in Mix‐n‐Stain Antibody Storage Buffer (Biotium).

PBS containing 1% ProLong Live Antifade Reagent (Thermo Fisher Scientific, P36975) was used as the imaging medium for immunofluorescence.

Immunofluorescence imaging of the cells was performed using a SpinSR10 (Olympus, Tokyo, Japan) with an oil‐immersion objective (Olympus, PlanApoN 60×/1.40 Oil). Fluorophores were excited at 405 nm (for Hoechst 33342 or CF405M), 488 nm (for Alexa Fluor 488), 512 nm (for mVenus), and 640 nm (for MitoTracker Deep Red FM).

### Mitochondria isolation

Mitochondria were isolated according to the instructions of the Mitochondria Isolation Kit for Cultured Cells (Thermo Fisher Scientific, 89874), except that the vortexing procedure was avoided to prevent fragmentation of TERT. 2.0 × 10^7^ cells were harvested and stored at −80 °C. Within 2 weeks, the cells were thawed and suspended in 4.8 mL of Reagent A in the kit supplemented with a proteinase inhibitor cocktail (100 mm 4‐(2‐aminoethyl) benzenesulfonyl fluoride, 2 mm leupeptin, 0.87 mm pepstatin, and 100 mm N‐p‐tosyl‐l‐arginine methyl ester in dimethyl sulfoxide). Following a 2‐min incubation on ice, the suspended cells were mixed with 60 μL of kit Reagent B and incubated on ice for 5 min with agitation by pipetting every minute. After mixed with 4.8 mL of kit Reagent C supplemented with the proteinase inhibitor cocktail, suspended cells were centrifuged at 700 × ***g*** for 10 min at 4 °C. The supernatant was transferred to new tubes and centrifuged at 3000 × ***g*** for 15 min at 4 °C. The pellet was mixed with 500 μL of the added Reagent C and centrifuged at 12 000 × ***g*** for 5 min at 4 °C. The pelleted mitochondria were washed with suspending the Reagent C and pelleting by centrifuge at 14 500 × ***g*** for 3 min at 4 °C for six times. Then, the pelleted cells were suspended directly in SDS/PAGE sample buffer (final 68 mm Tris–HCl, 2% SDS, 10% glycerine, 0.05% bromophenol blue, pH 6.8) and used for western blotting (Mitochondria). For further isolation of mitochondrial matrix proteins, the purified mitochondria were suspended in 100 mm sodium carbonate and incubated on ice for 30 min. After the addition of the protease inhibitor cocktail, the sample was ultracentrifuged at 75 000 × ***g*** for 40 min at 4 °C and the supernatant was collected (crude matrix). The whole cell extracts were prepared by suspending 5.0 × 10^6^ cells in cell lysis buffer (50 mm Tris–HCl, pH 8.0, 150 mm sodium chloride, 0.5% sodium deoxycholate, 0.1% sodium dodecyl sulfate, 1% NP‐40 substitute, 0.1 mm phenylmethylsulfonyl fluoride, 4.3 μm 2‐mercaptoethanol), incubating on ice for 30 min, and collecting the supernatant from a centrifugation at 16 000 × ***g***, 20 min, 4 °C.

### Dead cell detection with live‐cell imaging

Cells were cultivated for a week before the assay and passaged five times. A day before the assay, the cells were passaged into black‐walled poly‐l‐lysine coated 96‐well plates (Matsunami, GP96001) at a density of 1000 cells per well. All empty wells and space between wells were filled with sterilized water.

Cells were treated with DMEM supplemented with 10% FBS, 1 × PenStrep, and 267 μm sodium percarbonate (SPC, Fujifilm Wako Pure Chemical Corporation, equivalent to 267 μm sodium carbonate and 400 μm H_2_O_2_) for 3 h. We used SPC as oxidative stress treatment to make the concentration of hydrogen peroxide uniform in every experiment instead of hydrogen peroxide alone, which is unstable and may change its effective concentration among experiments. As negative controls, cells were treated with DMEM supplemented with 10% FBS, 1 × PenStrep, and 267 μm sodium carbonate. During imaging, all cells were cultured in DMEM supplemented with 10% FBS, 1 × PenStrep, 250 nM SYTOX Orange (Thermo Fisher Scientific, S11368) and 2.5 μm YO‐PRO‐1 (Thermo Fisher Scientific, Y3603) at 37 °C and 5% CO_2_.

For live‐cell imaging with Z‐VAD‐fmk, the cells were treated with DMEM supplemented with 10% FBS, 1 × PenStrep, 50 μm Z‐VAD‐fmk, and 267 μm SPC for 3 h. Then, the cells were cultured in DMEM supplemented with 10% FBS, 1 × PenStrep, 50 μm Z‐VAD‐fmk, 250 nm SYTOX Orange (Thermo Fisher Scientific) and 2.5 μm YO‐PRO‐1 (Thermo Fisher Scientific) at 37 °C and 5% CO_2_.

### Telomerase activity assay

5.0 × 10^6^ cells were harvested and frozen at −80 °C. Thawed cells were treated with cell lysis buffer in Telomerase Activity Quantification qPCR Assay Kit (ScienCell, Carlsbad, CA, USA, 8928) supplemented with 100 μm phenylmethylsulfonyl fluoride in isopropanol and 0.03% (v/v) β‐mercaptoethanol and assayed following the instructions of the kit. qPCR was performed using OneStepPlus (Thermo Fisher Scientific).

### Western blotting

The protein concentration of each lysate was measured using a Pierce 660 nm Protein Assay Kit (Thermo Fisher Scientific) on NanoDrop 2000c (Thermo Fisher Scientific). Nine or 10 μg of whole‐cell extracts prepared in telomerase activity assay, or 3 μg of isolated mitochondria was loaded on to a 4–15% precast polyacrylamide gel (Bio‐Rad, Hercules, CA, USA). Proteins were transferred onto a PVDF membrane using the Trans‐Blot Turbo Transfer System (Bio‐Rad) and the transferred membrane was blotted using the iBind Western System (Thermo Fisher Scientific). The primary antibodies used were anti‐TERT antibody (Rockland, 600‐401‐252S, dilution 1 : 200 or 1 : 500), anti‐β tubulin antibody (Cell Signaling Technology, 86298, dilution 1 : 200) anti‐COX IV antibody (Cell Signaling Technology, 4850, dilution 1 : 10 000), anti‐β actin antibody (Cell Signaling Technology, 8457, dilution 1 : 1000), anti‐Rpb1 NTD antibody (Cell Signaling Technology, 14958, dilution 1 : 500) and anti‐TFAM antibody (Cell Signaling Technology, 8076, dilution 1 : 500). IgG Detector Solution v2 from western blot Rapid v2 (Takara Bio) and Anti Rabbit‐IgG horseradish peroxidase (HRP)‐conjugated antibody (Cell Signaling Technology, 7074P2) were employed as the secondary antibody at a dilution of 1 : 1000 or 1 : 500. SuperSignal West Femto Maximum Sensitivity Substrate (Thermo Fisher Scientific) and ImmunoStar Basic (Fujifilm Wako Pure Chemical Corporation) were used as substrates for HRP. Protein bands were detected using an ImageQuant LAS4000 mini (GE Healthcare, Chicago, IL, USA), Amersham Imager 600 (GE Healthcare) or Nikon Z6 (Nikon, Tokyo, Japan), and the intensities were measured using the Gel tool of fiji/imagej [42].

### Mass spectroscopy

20 μg of isolated mitochondrial samples were loaded on to a 4–15% precast polyacrylamide gel (Bio‐Rad, Hercules, CA, USA). After electrophoresis at 200 V, 20 min, the gel were stained by EzStainAQua (ATTO, Tokyo, Japan). The bands corresponding to the ~ 120 kDa were cut out from the gel and analyzed using nanoLC‐MS/MS protein identification service (Nihon Proteomics, Miyagi, Japan). The results file was uploaded as a Supporting document.

### Dead cell detection and TERT visualization by live‐cell tracking

Cells were cultivated a week before the assay and passaged five times. A day before the assay, the cells were passaged into black‐walled poly‐l‐lysine coated 96‐well plates (Matsunami, GP96001) at a density of 1000 cells per well. All empty wells and space between wells were filled with sterilized water. Cells were incubated with DMEM supplemented with 10% FBS, 1 × PenStrep, and 100 nm MitoTracker Deep Red FM for 30 min at 37 °C and 5% CO_2_. For the oxidative stress, the cells were treated with DMEM supplemented with 10% FBS, 1 × PenStrep, and 267 μm SPC (equivalent to 267 μm sodium carbonate and 400 μm H_2_O_2_) for 3 h. As negative controls, cells were treated with DMEM supplemented with 10% FBS, 1 × PenStrep, and 267 μm sodium carbonate. During imaging, all cells were cultured in DMEM supplemented with 10% FBS, 1 × PenStrep and 250 nm SYTOX Blue (Thermo Fisher Scientific) at 37 °C and 5% CO_2_.

For control experiments with Hoechst 33342, cells were incubated with 10% FBS, 1 × PenStrep, and 100 nm MitoTracker Deep Red FM (Thermo Fisher Scientific) for 30 min at 37 °C and 5% CO_2_ and then were incubated with 10% FBS, 1 × PenStrep, and 20 μm Cellstain Hoechst 33342 (Fujifilm Wako Pure Chemical Corporation) for 15 min at 37 °C and 5% CO_2_.

### Microscopy for live‐cell imaging and tracking

Imaging was performed using an inverted microscope (Ti, Nikon) with an objective (Plan Apo Lambda 40×/0.95, Nikon), LED illumination system (X‐Cite XLED1, Lumen Dynamics, Ontario, Canada) and filter sets [CFP‐2432C (for SYTOX‐Blue or Cellstain Hoechst 33342; Semrock, Rochester, NY, USA), GFP‐3035D (for YO‐PRO‐1; Semrock), LF514‐B (for mVenus; Semrock), TRITC‐A‐Basic (for SYTOX‐Orange; Semrock), and Cy5‐4040C (for MitoTracker Deep Red FM; Semrock)]. Bright‐field and fluorescence images were captured using an electron multiplying CCD camera (ImagEM X2‐1K EM‐CCD, Hamamatsu Photonics, Shizuoka, Japan). The sample temperature was kept at 37 °C by feedback from a heat sensor in a water‐filled well and was monitored by NECO (Tokai Hit, Shizuoka, Japan).

For the live‐cell imaging, dead cells were defined as cells that were stained with SYTOX Orange or YO‐PRO‐1; the threshold was set to the mean intensity of negative control cells plus 10 times the standard deviation using NIS‐Elements software (Nikon).

For live‐cell tracking, a 1.5× magnification lens was used, and dead cells were defined as cells that were stained with SYTOX Blue; the threshold was set to the mean intensity of negative control cells plus 5 times the standard deviation using nis‐elements software (Nikon).

For control experiments with Hoechst 33342, dead cells were defined as cells that were stained with SYTOX Orange; the threshold was set to the mean intensity of negative control cells plus 10 times the standard deviation using nis‐elements software.

### Colocalization analysis

Each cell in each frame in a bright field image was used as a reference to select regions of interest manually. Otsu's method [43] was employed to find pixels above a certain fluorescence threshold and Manders' colocalization coefficients (MCCs) were calculated in the pixels using the Genome Damage Stability Centre (GDSC) colocalization plugins for fiji/imagej.

MCC of mVenus‐TERT with mitochondria was calculated from mVenus‐TERT intensity $T_{i}$ and mitochondria intensity $M_{i}$ of pixel i as:

$$\mathrm{MCC}=\frac{\sum_{i}T_{i,\text{colocal}}}{\sum_{i}T_{i}}$$

where $T_{i,\text{colocal}}$ = $T_{i}$ if $M_{i}$ > 0 and $T_{i,\text{colocal}}$ = 0 if $M_{i}$ = 0.

Kernel density estimation [44] was performed on MCC histograms in Fig. 5A and Figs S5, S6 and S8B. Kernel density estimation estimates the true probability density function from the dataset, which corresponds to MCC distributions in this work.

### Statistical analysis

Statistical tests were performed using r software (R Foundation for Statistical Computing, Vienna, Austria) and python software (Python Software Foundation, Wilmington, DE, USA). The Steel‐Dwass test was performed in Figs 1B and 3B, and Figs S7C and S8D,E using the NSM3 library in R with the Monte Carlo method. The Mann–Whitney *U*‐test was performed in Fig. 3D. The pairwise logrank test was performed in Fig. S8A. The Tukey's multiple comparison test on proportions was performed in Fig. S8B.

## Conflict of interest

The authors declare no conflict of interest.

## Author contributions

HE and TS contributed to conceptualization, methodology, and investigation; HE contributed to analysis; HE, TS, and SU contributed to writing—original draft; HE, TS, RI, and SU contributed to draft editing.

## Supporting information


**Fig. S1.** TERT protein level in mitochondrial fraction and whole‐cell extracts.
**Fig. S2.** Survival curves of YO‐PRO‐1 after oxidative stress and of SYTOX Orange and YO‐PRO1 without oxidative stress.
**Fig. S3.**TERT protein level for mVenus‐TERT in whole‐cell extracts.
**Fig. S4.** Imaging with 267 μm sodium carbonate does not show cytotoxicity.
**Fig. S5.** Cells without oxidative stress do not show high MCC of mVenus‐TERT with mitochondria.
**Fig. S6.** Dead cells do not show high MCC of Hoechst 33342 with mitochondria after oxidative stress.
**Fig. S7.** Dead cells after oxidative stress show a non‐significantly decreased expression of TERT.
**Fig. S8.** TERT_R3E/R6E_ and TERT_Y707F_ mutants display decreased apoptosis of cells with low MCC and show no correlation between the initial MCC and time until cell apoptosis.
**Table S1.** Key PCR primers used to generate the TERT constructs in this study.

## Data Availability

The authors declare that the datasets used and/or analyzed during the current study are available from the corresponding author on reasonable request. Raw images of western blotting and mass spectrometry data are uploaded as supporting documents of this paper.
