# Supplementary material for: Accumulation of TERT in mitochondria exerts two opposing effects on apoptosis
Source: FEBS Open Bio. 2023 Aug 8;13(9):1667–82. doi: 10.1002/2211-5463.13682 (PMC10476567; doi:10.1002/2211-5463.13682)
Supplement: Supplementary file 1 — Fig. S1. TERT protein level in mitochondrial fraction and whole‐cell extracts. Fig. S2. Survival curves of YO‐PRO‐1 after oxidative stress and of SYTOX Orange and YO‐PRO1 without oxidative stress. Fig. S3.TERT protein level for mVenus‐TERT in whole‐cell extracts. Fig. S4. Imaging with 267 μm sodium carbonate does not show cytotoxicity. Fig. S5. Cells without oxidative stress do not show high MCC of mVenus‐TERT with mitochondria. Fig. S6. Dead cells do not show high MCC of Hoechst 33342 with mitochondria after oxidative stress. Fig. S7. Dead cells after oxidative stress show a non‐significantly decreased expression of TERT. Fig. S8. TERTR3E/R6E and TERTY707F mutants display decreased apoptosis of cells with low MCC and show no correlation between the initial MCC and time until cell apoptosis. Table S1. Key PCR primers used to generate the TERT constructs in this study. [file FEB4-13-1667-s001.pdf]

Supplementary Information

Supplementary Figure S1

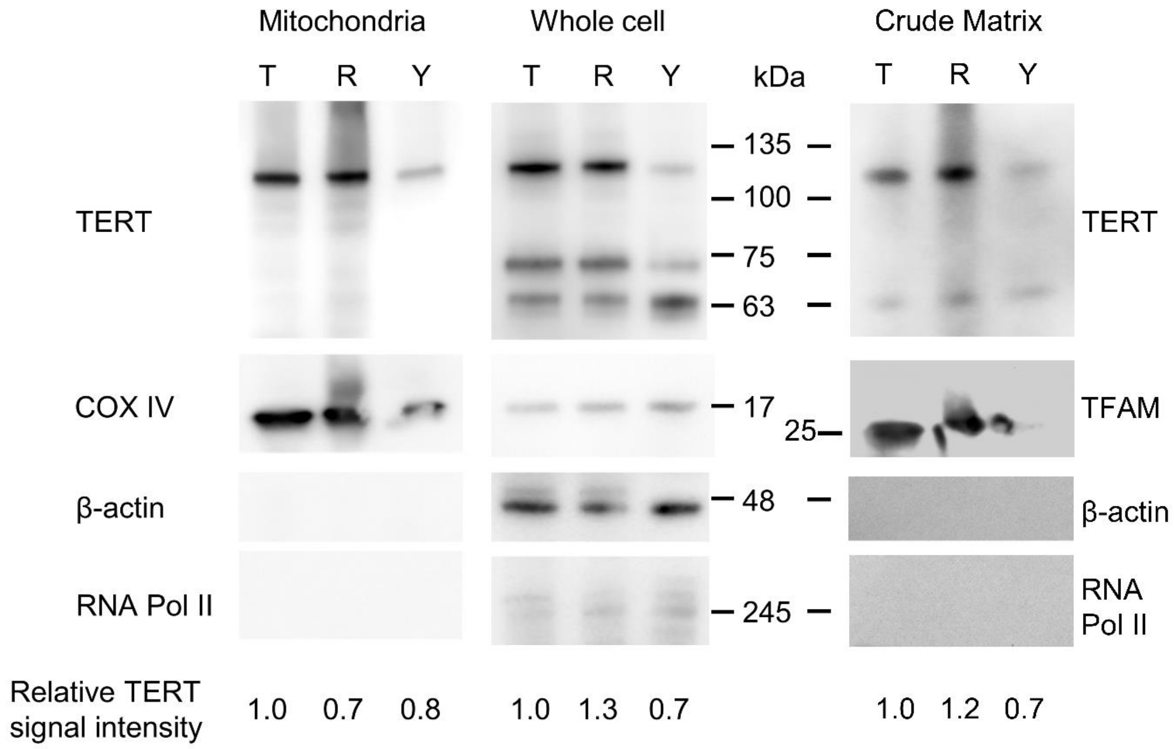

Supplementary Figure S1. TERT protein level in mitochondrial fraction and whole-cell extracts.

Representative images of western blotting of isolated mitochondria (left panels), whole cell extracts (middle panels) and crude matrix (right panels) from the cells expressing $TERT_{WT}$ (T), $TERT_{R3E/R6E}$ (R), and $TERT_{Y707F}$ (Y). Expected molecular weight for each protein is: TERT, 120 kDa; COX IV, 17 kDa; β-actin, 42 kDa, RNA Polymerase II (RNA Pol II), 250 kDa. The isolation of the mitochondrial fraction was confirmed by the presence of the mitochondrial

marker COX IV and the absence of the nuclear marker RNA Pol II and the cytosolic marker β-actin, both of which were present in the whole-cell extracts. COX IV and β-actin were used as a loading control for the mitochondrial fraction and the whole-cell extracts, respectively. The amount of TERT protein was quantified from the 63–135 kDa bands that appeared when stained with anti-TERT antibody alone. The TERT protein signal intensities were then divided by the intensities of the loading controls (COX IV for mitochondrial fraction and β-actin for whole-cell extracts) to evaluate the TERT protein level of each sample as relative TERT signal intensity. From this mitochondrial sample, the membrane fraction was further removed by sodium carbonate treatment and ultracentrifugation to determine the amount of TERT present in the matrix. In this case, the TERT signal intensities were divided by the intensities of the transcription factor A, which is a matrix loading control (TFAM).

Supplementary Figure S2

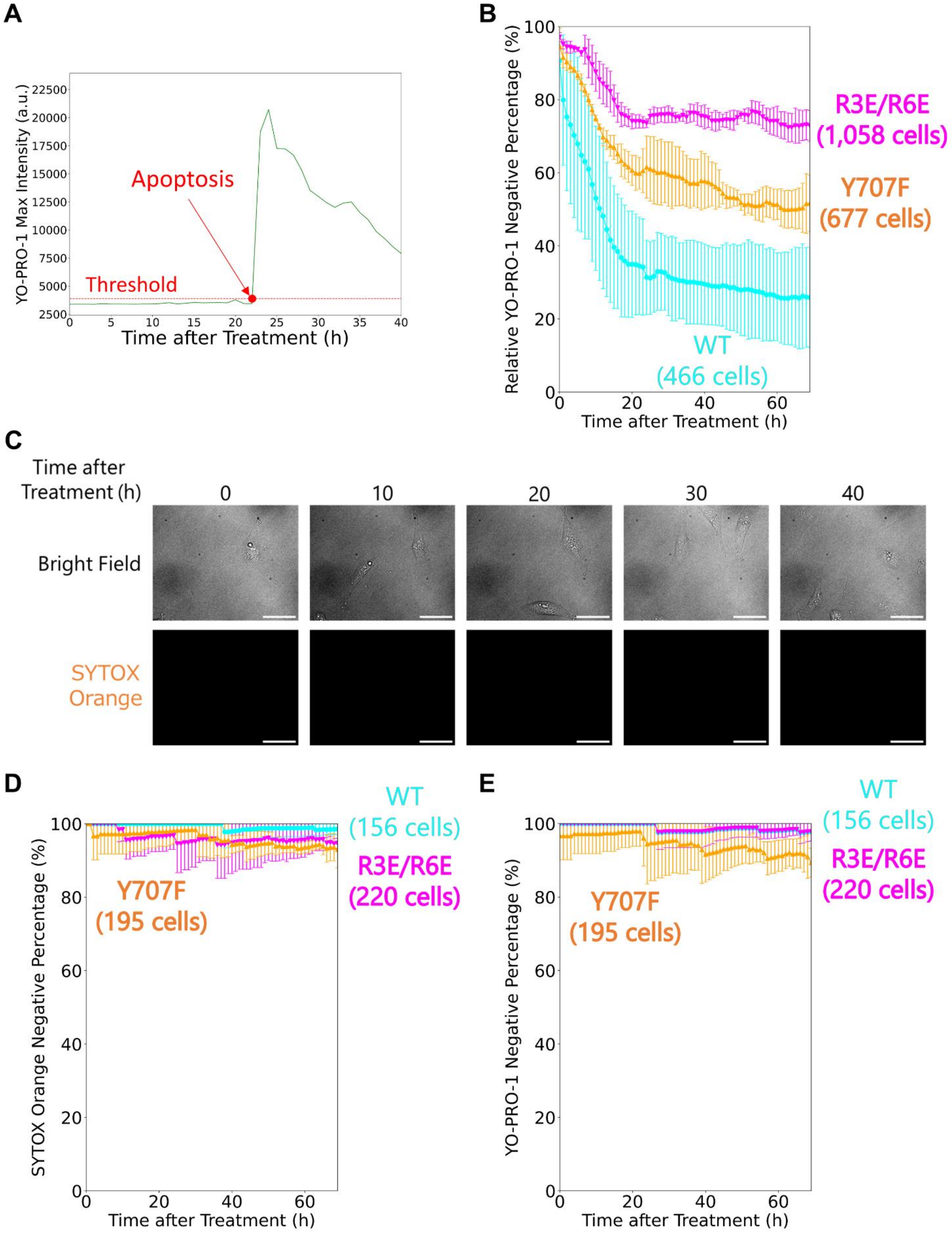

Supplementary Figure S2. Survival curves of YO-PRO-1 after oxidative stress and of SYTOX Orange and YO-PRO1 without oxidative stress.

A. A representative time-course trace of the maximum fluorescence intensity of the apoptotic cell staining dye YO-PRO-1 from the cell depicted in Fig. 2A. **B.** Percentage of YO-PRO-1-negative cells treated with 267 μM SPC for 3 h. All percentages were normalized to the data in Supplementary Fig. S2E. Error bars show 95% C.I. (1.96 SEM) from 3 independent experiments. WT, 466 cells; R3E/R6E, 1,058 cells; Y707F, 677 cells. **C.** Representative live-cell images of HeLa cells expressing the TERT constructs. Cells were treated with 267 μM sodium carbonate for 3 h before imaging (no oxidative stress). Orange, SYTOX Orange fluorescence. Scale bars, 50 μm. **D**. Percentage of SYTOX Orange-negative cells treated with 267 μM sodium carbonate for 3 h before imaging (no oxidative stress). Error bars show 95% C.I. (1.96 SEM) from 3 independent experiments. WT, 156 cells; R3E/R6E, 220 cells; Y707F, 195 cells. **E**. Percentage of YO-PRO-1-negative cells treated with 267 μM sodium carbonate for 3 h before imaging (no oxidative stress). Error bars show 95% C.I. (1.96 SEM) from 3 independent experiments. WT, 156 cells; R3E/R6E, 220 cells; Y707F, 195 cells.

Supplementary Figure S3

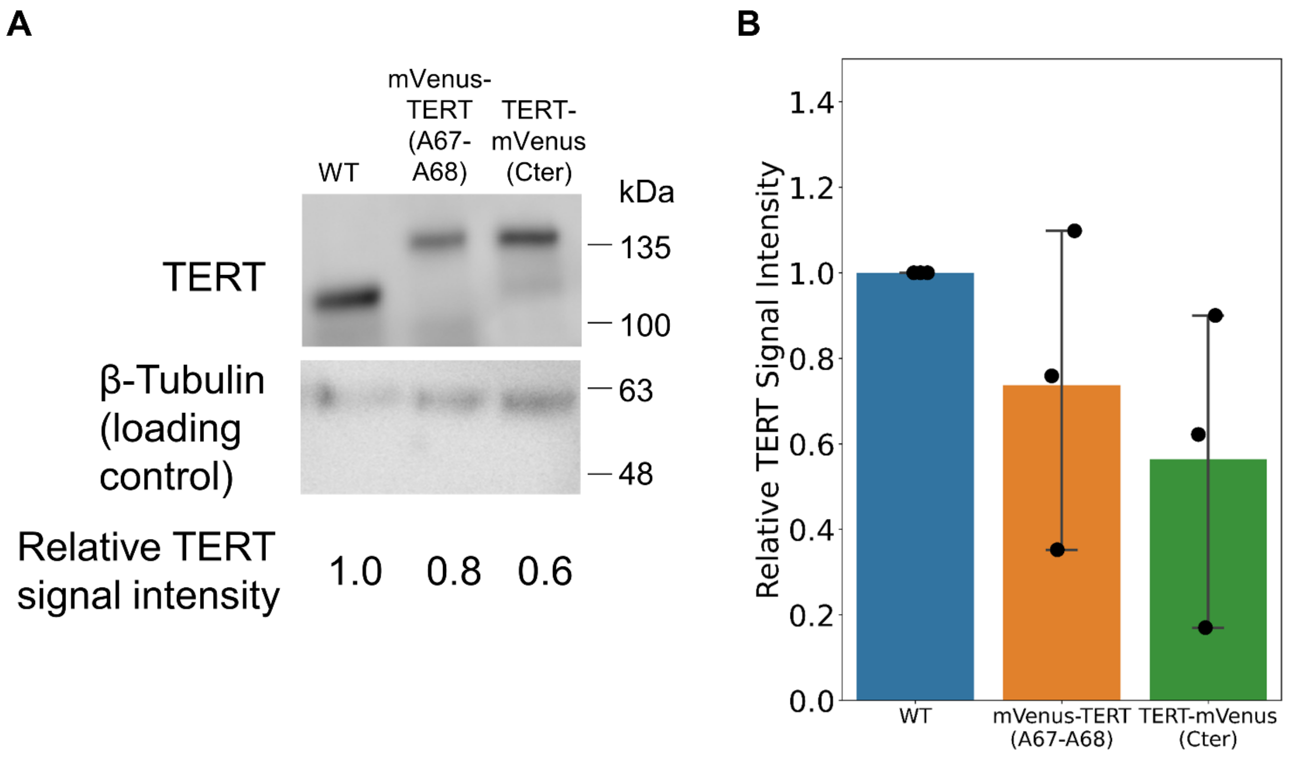

Supplementary Figure S3. TERT protein level for mVenus-TERT in whole-cell extracts.

A. Representative images of western blotting of whole-cell extracts. TERT-mVenus (Cter) is a TERT construct conjugated with mVenus at its C-terminus. β-tubulin served as a loading control. **B.** TERT signal intensity from western blotting of whole-cell extracts. Dots show individual data points and bars show the mean ± 95% C.I. (1.96 SEM) from 3 independent experiments. Steel-Dwass test was performed with a significance level of 0.05.

Supplementary Figure S4

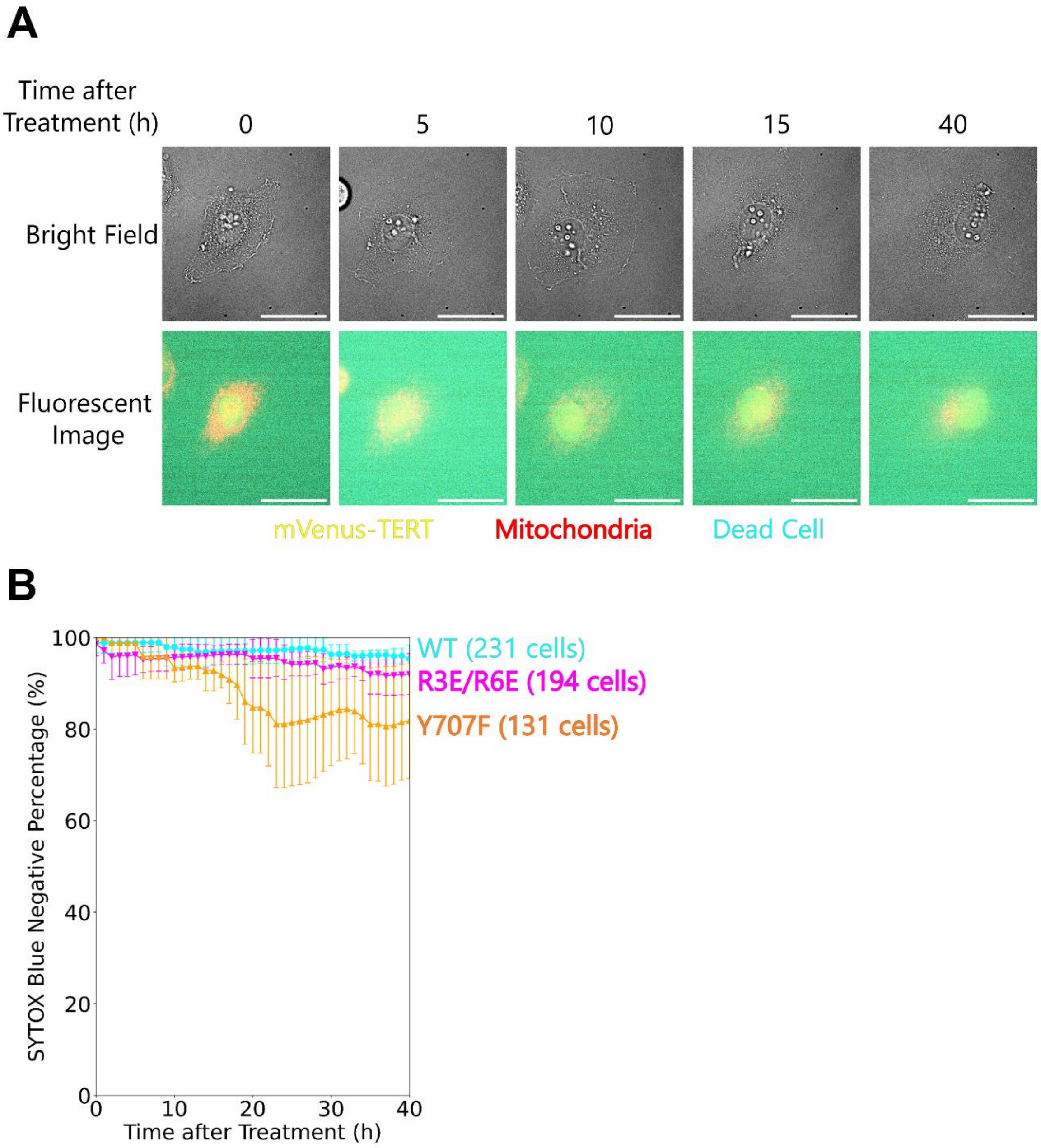

Supplementary Figure S4. Imaging with 267 μM sodium carbonate does not show cytotoxicity.

A. Representative live-cell images of HeLa cells expressing mVenus-TERT constructs treated with 267 μM sodium carbonate for 3 h before the imaging (no oxidative stress). Yellow,

mVenus fluorescence; red, MitoTracker Deep Red FM fluorescence; cyan, SYTOX Blue fluorescence. Scale bars, 50 μm. **B.** Percentage of SYTOX Blue-negative cells treated with 267 μM sodium carbonate for 3 h before imaging (no oxidative stress). Error bars show 95% C.I. (1.96 SEM) from 3 independent experiments. mVenus-$TERT_{WT}$ (WT), 231 cells; mVenus-$TERT_{R3E/R6E}$ (R3E/R6E), 194 cells; mVenus-$TERT_{Y707F}$ (Y707F), 131 cells.

Supplementary Figure S5

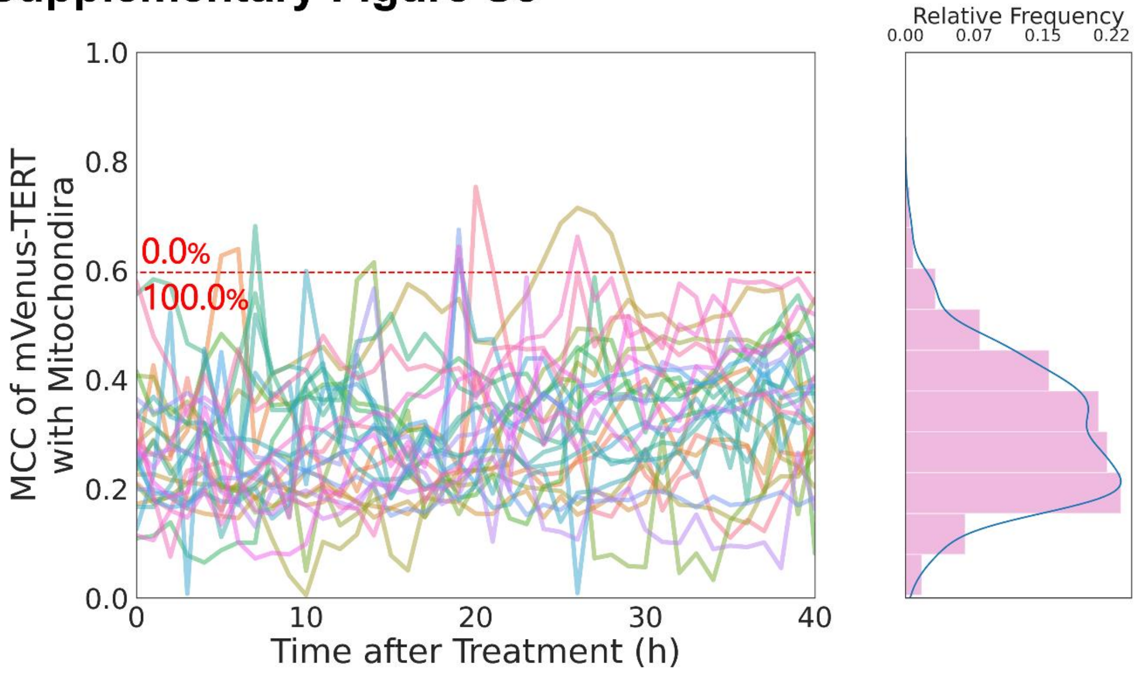

Supplementary Figure S5. Cells without oxidative stress do not show high MCC of mVenus-TERT with mitochondria.

Time-course plot of MCC of mVenus-TERT with mitochondria in each cell treated with 267 μM sodium carbonate for 3 h before the imaging (no oxidative stress). All cells here survived during imaging. Plotted values are the mean of values per 5 frames. Red numbers show the percentage of cells whose initial MCC of mVenus-TERT with mitochondria was above or below the threshold represented by the red dashed line. A histogram and KDE plot of all MCCs are shown in the right panel. Total 27 cells.

Supplementary Figure S6

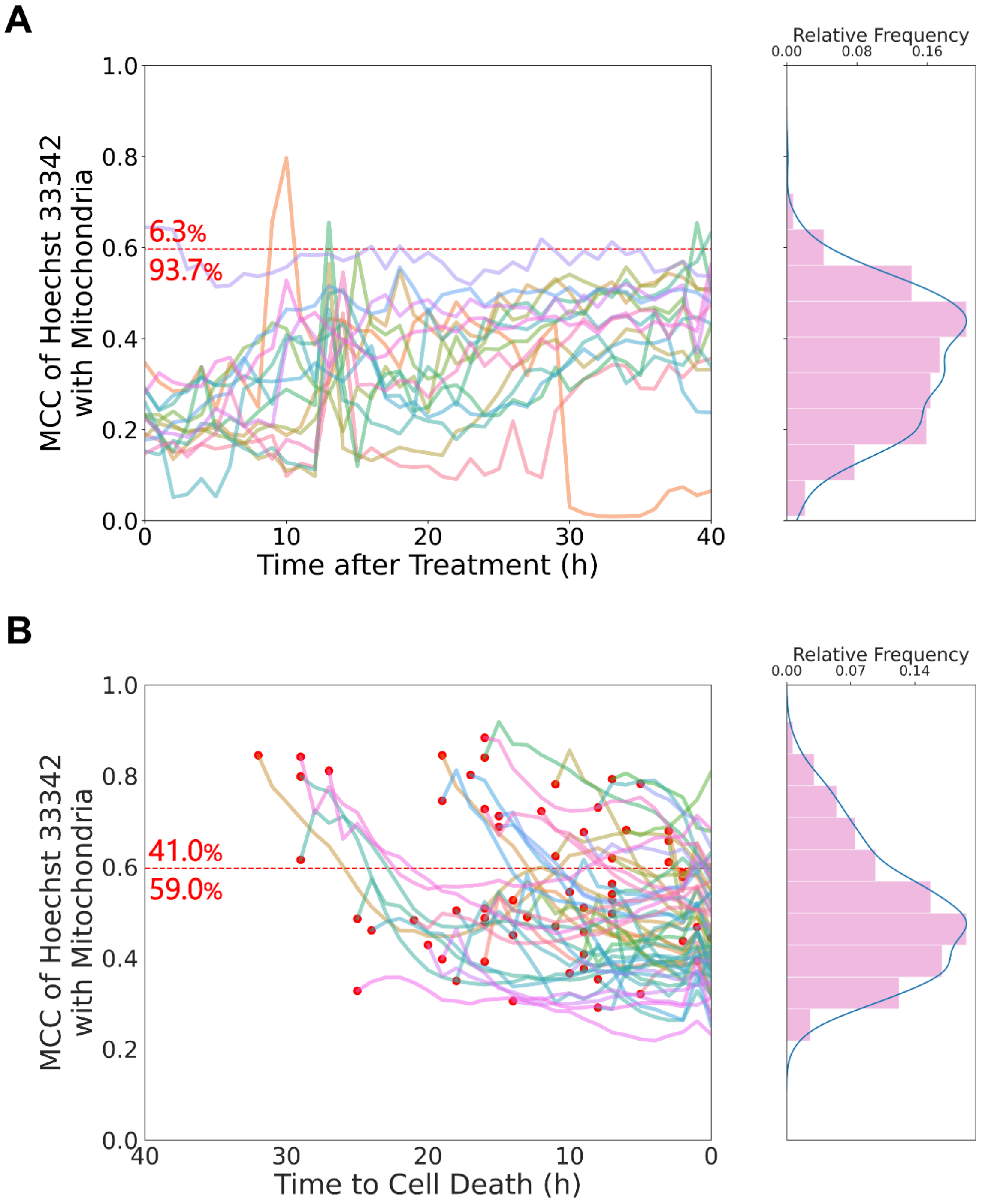

Supplementary Figure S6. Dead cells do not show high MCC of Hoechst 33342 with mitochondria after oxidative stress.

A. Time-course plot of MCC of Hoechst 33342 with mitochondria in each cell treated with 267 μM sodium carbonate for 3 h before the imaging (no oxidative stress). All cells here survived during imaging. Plotted values are the mean of values per 5 frames. Red numbers show the percentage of cells whose initial MCC of mVenus-TERT with mitochondria was above or below the threshold represented by the red dashed line. A histogram and KDE plot of all MCCs are shown in the right panel. Total 16 cells. **B**. Time-course plot of MCC of Hoechst 33342 with mitochondria of cells treated with 267 μM SPC for 3 h, in which all cells died. Plotted values are the mean of values per 5 frames. 0 in the x-axis represents the moment the cells died. Red dots show the MCC of Hoechst 33342 with mitochondria at the beginning of the observation. Red numbers show the percentage of cells whose initial MCC of mVenus-TERT with mitochondria was above or below the threshold represented by the red dashed line. A histogram and KDE plot of the MCC values are shown in the right panel. Total 61 cells.

Supplementary Figure S7

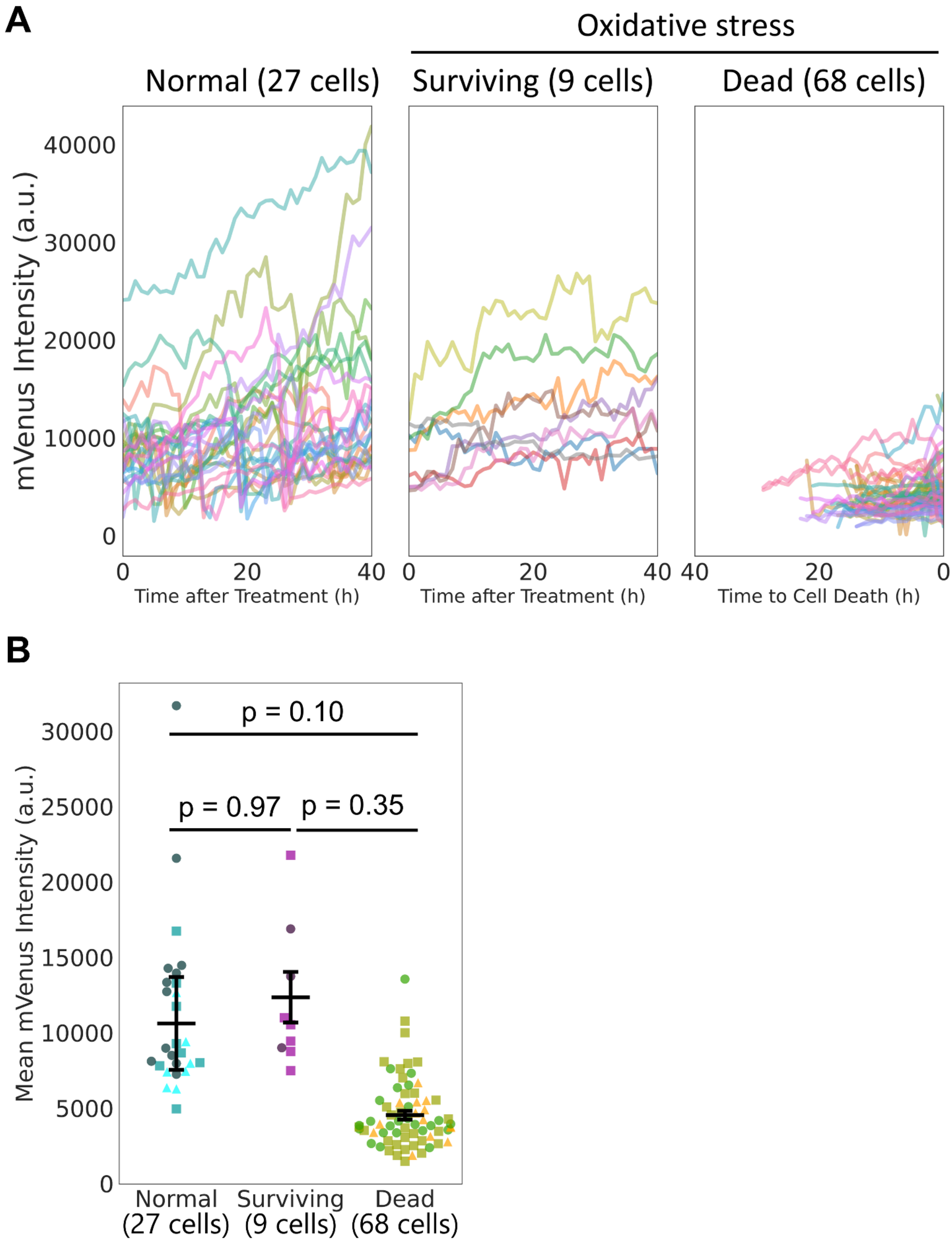

Supplementary Figure S7. Dead cells after oxidative stress show a non-significantly decreased expression of TERT.

A. Time-course plot of mVenus intensity in each cell. As oxidative stress, we treated cells with 267 μM SPC for 3 h. For dead cells, 0 in the x-axis represents the moment the cells died. All cells under normal conditions (without oxidative stress) here survived during imaging. Normal cells (Normal), 27 cells; Surviving cells (Surviving), 9 cells; Dead cells (Dead), 68 cells.

B. Quantification of the mean mVenus intensity in each cell. Dots show the mean mVenus intensity of each cell and bars show the mean ± 95% C.I. (1.96 SEM) from 3 independent experiments. Different markers represent different experiments. Normal cells (Normal), 27 cells; Surviving cells (Surviving), 9 cells; Dead cells (Dead), 68 cells. Steel-Dwass test was performed with a significance level of 0.05.

Supplementary Figure S8

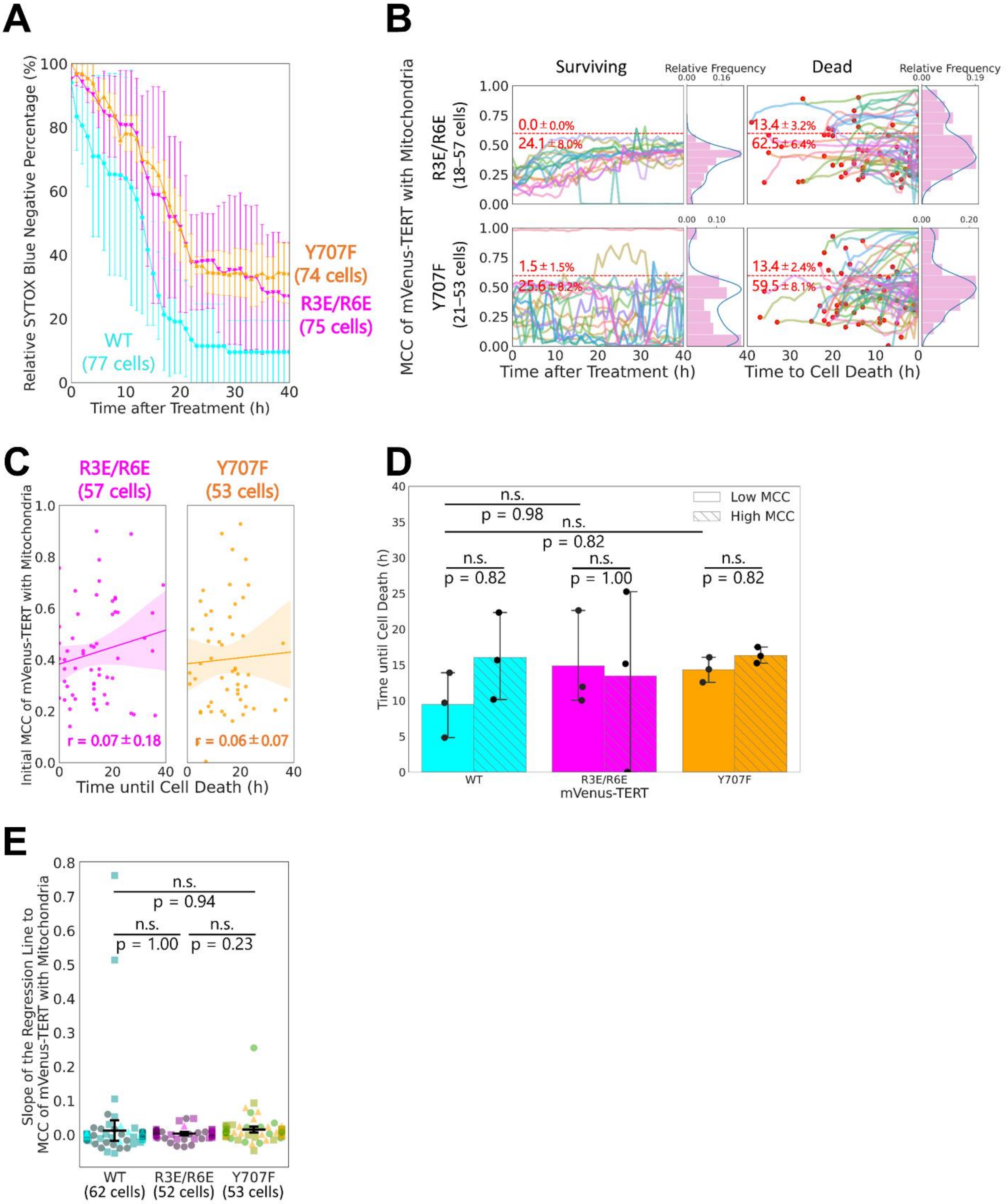

Supplementary Figure S8. $TERT_{R3E/R6E}$ and $TERT_{Y707F}$ mutants display decreased the apoptosis of cells with low MCC and show no correlation between the initial MCC and time until cell apoptosis.

A. Percentage of SYTOX Blue-negative cells treated with 267 μM SPC for 3 h. All percentages were normalized to control experiments without oxidative stress (Supplementary Fig. S4B). Error bars show 95% C.I. (1.96 SEM) from 3 independent experiments. mVenus-$TERT_{WT}$ (WT), 77 cells; mVenus-$TERT_{R3E/R6E}$ (R3E/R6E), 75 cells; mVenus-$TERT_{Y707F}$ (Y707F), 74 cells. Pairwise logrank test with Bonferroni's correction for all the cells was performed with a significance level of 0.01 and showed statistical significance with $p < 0.005$ for WT–R3E/R6E and $p < 0.005$ for WT–Y707F. **B.** Time-course plot of MCC of mVenus-TERT with mitochondria in each cell in Supplementary Fig. S8A. A histogram and KDE plot of all MCCs are shown. Plotted values are the mean of values per 5 frames. For dead cells, the beginning of cell death was set to 0 in the x-axis. Red dots show MCC of mVenus-TERT with mitochondria at the beginning of the observation. Red numbers show the percentage (mean ± SEM from 3 independent experiments) of cells whose initial MCC of mVenus-TERT with mitochondria was above or below the threshold represented by the red dashed line. For mVenus-$TERT_{R3E/R6E}$ (R3E/R6E), surviving cells (Surviving), 18 cells; Dead cells (Dead), 57 cells. For mVenus-$TERT_{Y707F}$ (Y707F),

surviving cells (Surviving), 21 cells; Dead cells (Dead), 53 cells. Tukey's multiple comparison test on proportions of dead cells having low MCC between the cell lines was performed with a significance level of 0.05 and the differences did not reach statistical significance ($p = 0.06$ for WT–R3E/R6E, $p = 0.05$ for WT–Y707F). **C.** Scatter plot and regression line between the initial MCC of mVenus-TERT with mitochondria of each dead cell and time until cell death in Supplementary Fig. S8B. Translucent bands around the regression line represent 95% C.I. (1.96 SEM). r, Pearson's correlation coefficient (PCC) shows the mean ± SEM from 3 independent experiments. mVenus-TERT$_{R3E/R6E}$ (R3E/R6E), 57 cells; mVenus-TERT$_{Y707F}$ (Y707F), 53 cells. **D.** Time until death of cells with high or low initial MCC of mVenus-TERT with mitochondria in Fig. 5A and Supplementary Fig. S8B. Graphs show data from each cell and the mean ± 95% C.I. (1.96 SEM) from 3 independent experiments. Steel-Dwass test was performed with a significance level of 0.05 and no statistical significance was detected. **E.** Slope of a regression line of MCC of mVenus-TERT with mitochondria in Supplementary Fig. S8B. The regression lines were obtained by line-fitting of the time-course plots of MCC in Fig. 5A and Supplementary Fig. S8B. Dots show the slope of each dead cell and bars show the mean ± 95% C.I. (1.96 SEM) from 3 independent experiments. Different markers represent different experimental replicates. mVenus-TERT$_{WT}$ (WT), 62 cells; mVenus-TERT$_{R3E/R6E}$

(R3E/R6E), 52 cells; mVenus-TERT$_{Y707F}$ (Y707F), 53 cells. Steel-Dwass test was performed with a significance level of 0.05.

Supplementary Table S1. Key PCR primers used to generate the TERT constructs in this study.

Construct	PCR Template	PCR Primer
TERT	pCDH-3xFLAG-TERT	5′-AAACTACCCCAAGCTGGCCTCTGAGGCCATGC-3′<br>5′-TTGATCCCCAAGCTTGGCCTGACAGGCCTCAG-3′
mVenus	pCS2-mVenus	5′-ATGGTGAGCAAGGGCGAGG-3′<br>5′-CTTGTACAGCTCGTCCATGCCG-3′
pSBbi-TERT R3E/R6E-Pur	pSBbi-TERT-Pur	5′-CCCGAGTGCCGAGCCGTGCGCTCCCTG-3′<br>5′-AGCCTCCGGCATGGCCTCAGAGGCCTTTCGA-3′
pSBbi-TERT Y707F-Pur	pSBbi-TERT-Pur	5′-TTTGTCAAGGTGGATGTGACGG-3′<br>5′-GAACAGCTCAGGCGGCG-3′
pSBbi-mVenus-TERT R3E/R6E-Pur	pSBbi-mVenus-TERT-Pur	5′-CCCGAGTGCCGAGCCGTGCGCTCCCTG-3′<br>5′-AGCCTCCGGCATGGCCTCAGAGGCCTTTCGA-3′
pSBbi-mVenus-TERT Y707F-Pur	pSBbi-mVenus-TERT-Pur	5′-TTTGTCAAGGTGGATGTGACGG-3′<br>5′-GAACAGCTCAGGCGGCG-3′

Construct	PCR Template	PCR Primers	Inserts
pSBbi-TERT-Pur	pSBbi-Pur	5′-AAGCTTGGGGATCAATTCTCTAGAG-3′<br>5′-AGCTTGGGGTAGTTTTCACGAC-3′	TERT
pSBbi-mVenus-TERT-Pur (A67-A68)	pSBbi-TERT-Pur	5′-GACGAGCTGTACAAGGCCCCCTCCTTCCGCCA-3′<br>5′-GCCCTTGCTCACCATGGCGGGGGGCGGCCGT-3′	mVenus
pSBbi-TERT-mVenus-Pur (Cter)	pSBbi-TERT-Pur	5′-GACGAGCTGTACAAGTGAGGCCTGTCAG GCC-3′<br>5′-CTCGCCCTTGCTCACCCCCGGACCCGGAC CGTCCAGGATGGTCTTGAAGTCTG-3′	mVenus
